# Synergic Deprotonation Generates Alkali‐Metal Salts of Tethered Fluorenide‐NHC Ligands Co‐Complexed to Alkali‐Metal Amides

**DOI:** 10.1002/chem.201806278

**Published:** 2019-02-19

**Authors:** Kieren J. Evans, Stephen M. Mansell

**Affiliations:** ^1^ Institute of Chemical Sciences Heriot-Watt University Edinburgh EH14 4AS UK

**Keywords:** alkali metals, bimetallic base, carbene ligands, metalation

## Abstract

Synergic combinations of alkali‐metal hydrocarbyl/amide reagents were used to synthesise saturated N‐heterocyclic carbene (NHC) ligands tethered to a fluorenide anion through deprotonation of a spirocyclic precursor, whereas conventional bases were not successful. The Li_2_ derivatives displayed a bridging amide between two Li atoms within the fluorenide‐NHC pocket, whereas the Na_2_ and K_2_ analogues displayed extended solid‐state structures with the fluorenide‐NHC ligand chelating one alkali metal centre.

Many recent studies of bimetallic bases have shown synergic behaviour that gives them enhanced reactivity and selectivity in deprotonation reactions over more conventional bases.^1^ Highlights include the tetrametalation of ferrocene with a Na/Mg amide rather than the more usual mono‐ or di‐metalation with *n*BuLi and tetramethylethylenediamine (TMEDA),^2^ the stabilization of the reactive THF anion,^3^ and the selective *meta*‐metalation of toluene rather than at the methyl group or other aryl positions.^4^ A variety of s‐block‐metal NHC complexes have been reported,^5^ with bimetallic examples usually derived from: i) the functionalisation of a free carbene,^6^ ii) the deprotonation of the imidazol(in)ium salt,^7^ or iii) the reaction of preformed metal NHC complexes.^8^

Tethered NHC complexes consist of an NHC covalently linked to another donor, either neutral (L) or charged (X).^9^ X‐type examples have typically used alkali‐metal NHC salts as precursors to enable coordination to the targeted metal.^10^ In the system investigated by Danopoulos and co‐workers, single deprotonation of a tethered imidazolium salt afforded a neutral species **I** that existed in equilibrium with the zwitterionic species **II** (Scheme 1 a).10a A further equivalent of base then afforded the desired NHC‐fluorenide salt.^11^ Potassium salts of alkoxy‐carbenes with an unsaturated backbone could also be readily formed from the parent imidazolium salt with an excess of KH.5b In contrast, the analogous saturated alkoxy‐carbene system (Scheme 1 b) displayed different behaviour in which spirocyclic **III** was formed with one equivalent of base, and it was then ring‐opened with strongly basic rare‐earth salts.^12^

**Scheme 1:**
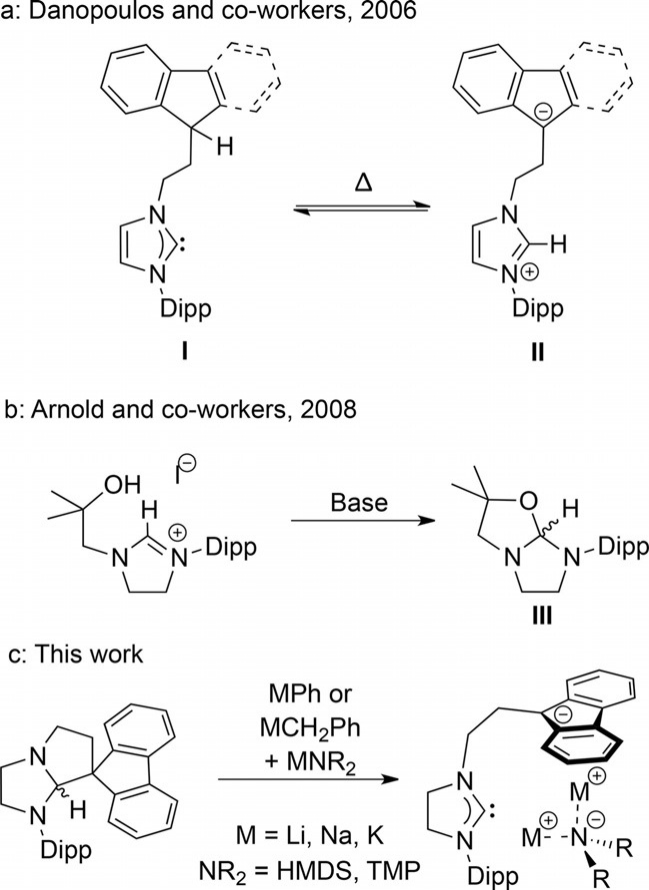
Observed isomers for selected functionalised NHCs, Dipp=2,6‐diisopropylphenyl.

Previously, we have used diamino‐fluorene precursors to generate saturated N‐heterocyclic stannylenes (NHSns) with a fluorenyl tether,^13^ and we recognised that the corresponding saturated NHCs could be accessible from the same precursor. Herein, we describe that synergic bimetallic bases are required to generate fluorenide‐tethered saturated NHCs, forming bimetallic complexes with the alkali‐metal amide in the process (Scheme 1 c).

Imidazolinium salt **1** was synthesised by orthoformate cyclisation using NH_4_BF_4_ from a previously reported diamine^13^ (Scheme 2, see the Supporting Information for the molecular structure).^14^ In contrast to the reactivity found for the unsaturated analogues,10a reaction of imidazolinium salt **1** with *n*BuLi yielded spirocycle **2** as a colourless solid.^12^ Compound **2** was found to be highly soluble in organic solvents and was recrystallised from a saturated petroleum‐ether solution (see the Supporting Information for the molecular structure). ^1^H NMR spectroscopy reveals a distinctive singlet at 5.45 ppm for the imidazoline H atom, and all four CH_3_ groups on the 2,6‐diisopropylphenyl (Dipp) substituent are inequivalent in **2**. The cyclisation is similar to that observed for saturated alkoxy‐carbenes.12a This reactivity is related to the differing electronics between saturated and unsaturated NHCs, suggesting that saturated NHCs act as better nucleophiles and electrophiles.^15^ Although the alkoxy‐carbene adduct **III** could be ring‐opened with strong bases, attempts to do so with **2** using *n*BuLi or KCH_2_Ph were unsuccessful. Even the use of *n*BuLi/TMEDA gave minimal conversion to the desired product, whereas Schlosser's base (*n*BuLi/KO*t*Bu)^16^ led to a mixture of products.

**Scheme 2:**
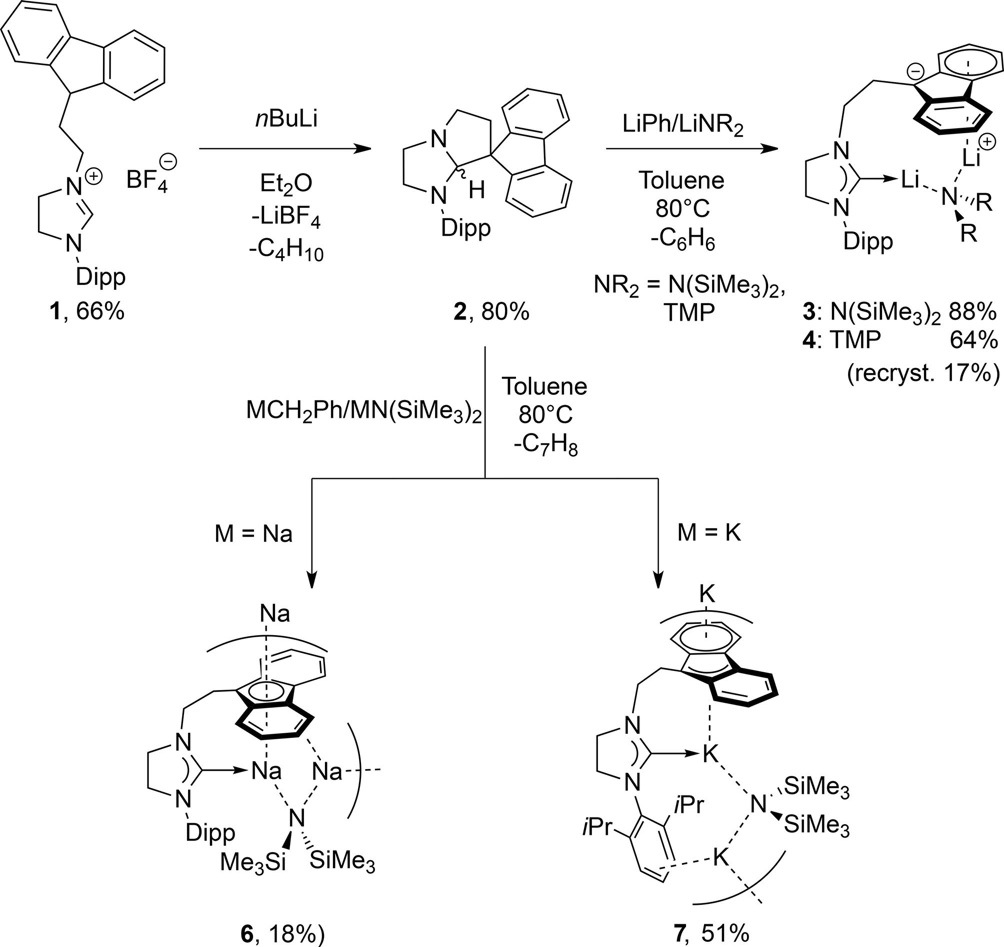
Generation and reactivity of spirocycle **2** with bimetallic reagents. Dipp=2,6‐*i*Pr_2_C_6_H_3_; TMP=2,2,6,6‐tetramethylpiperidide.

Previously in our group, we characterised a LiPh–LiTMP adduct (TMP=2,2,6,6‐tetramethylpiperidide),^17^ which inspired us to try reactions of the spirocycle with potentially synergic 1:1 mixtures of LiPh/LiNR_2_ [NR_2_=N(SiMe_3_)_2_, TMP]. These reactions successfully generated NHC‐dilithium complexes **3** and **4**, respectively, as red‐orange crystals, featuring a bridging amide situated between two Li cations that occupy the fluorenyl‐carbene pocket (**3**: Figure 1, **4**: see Supporting Information). The Li–carbene bond lengths are identical within error [**3**: 2.109(3), **4**: 2.118(3) Å], and similar to those found in the literature.6c, 7d Unsolvated [Li_2_(fluorenide)_2_] also has η^6^‐interactions,^18^ but the coordination geometry of fluorenide is relatively flexible.^19^ The Li−N distances for **3** and **4** are similar [**3**: Li1−N3 1.949(3), Li2−N3 1.967(3) Å; **4**: Li1−N3 1.944(3), Li2−N3 1.929(3) Å]. ^7^Li NMR spectroscopy of C_6_D_6_ solutions of **3** showed two distinctive resonances for the Li coordinated to the NHC at −0.87 ppm and the Li coordinated to the fluorenyl at −5.69 ppm due to aromatic ring currents from the fluorenyl system. The corresponding TMP complex **4** has similar chemical shifts of 0.09 and −5.40 ppm, respectively. The reaction of **2** with 0.5 equivalents of the previously reported aggregate [(LiTMP)_2_LiPh]_2_ was also found to afford **4**. It has been observed that strong bases (such as LiCH_2_SiMe_3_) can degrade saturated NHCs;5h no such degradation was observed in our case, even upon heating.

**Figure 1:**
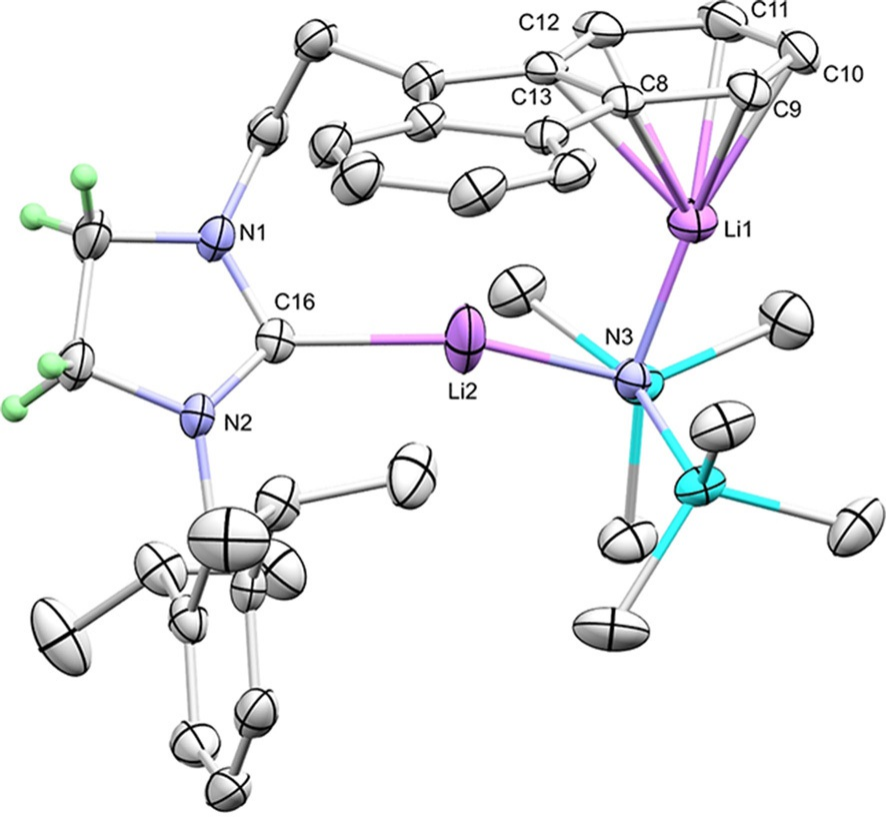
Molecular structure of **3** (thermal ellipsoids at 50 %). For clarity, only selected H atoms are shown.

Further investigation of the mixture of LiPh and LiN(SiMe_3_)_2_ showed good solubility in toluene or benzene whereas pure LiPh is not soluble. From a concentrated solution, we isolated a 1:1 adduct of LiPh/LiN(SiMe_3_)_2_ (**5**) (Scheme 3 and Figure 2). Unlike the LiPh/LiTMP adduct,^17^ **5** was formed at ambient temperature within five minutes. The X‐ray‐determined structure is similar to that found for LiPh by powder diffraction^20^ with a Li_2_X_2_ [X=μ^2^‐Ph, μ^2^‐N(SiMe_3_)_2_] core, which then stacks with neighbouring Ph rings to form an extended polymeric structure. The Li−C1 distances in **5** are symmetrical [2.204(3) Å] and shorter than those in Li_2_Ph_2_ [2.242(14) and 2.322(14) Å],^20^ whereas the close contacts to the neighbouring phenyl ring [η^3^: 2.527(2)–2.679(2) Å] are longer than in [Li_2_Ph_2_]_*n*_ [*ipso* and *ortho* C: 2.401(12)–2.534(14) Å, *meta* and *para* C: 2.745(15)–2.862(14)].^20^ Li η^3^‐Ph interactions are already known in the literature.^21^ The weaker interactions between Li_2_X_2_ units and the presence of SiMe_3_ groups may explain the greatly increased solubility of **5** in aromatic solvents compared with LiPh.

**Scheme 3:**
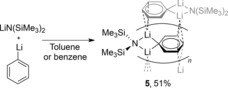
Formation of **5** from a 1:1 mixture of LiPh and LiN(SiMe_3_)_2_.

**Figure 2:**
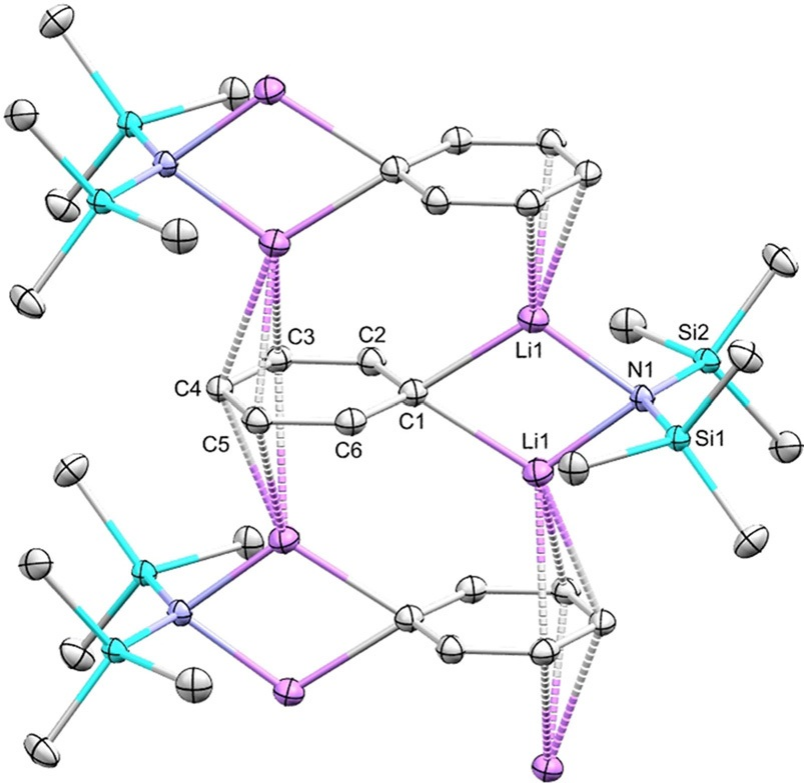
Molecular structure of LiPh/LiN(SiMe_3_)_2_ **5** (thermal ellipsoids at 50 %, H atoms omitted for clarity) displayed with a representation of the extended structure indicated. Selected distances [Å] and angles [°]: C1−Li1 2.204(3), Li1−N1 2.010(2), Li1−C4′ 2.527(2); Li1‐C1‐Li1 67.44(12), C1‐Li1‐N1 108.80(9).

Moving to the heavier Group 1 analogues, reaction of **2** with either 1:1 NaCH_2_Ph/NaN(SiMe_3_)_2_ or 1:1 KCH_2_Ph/KN(SiMe_3_)_2_ yielded very poorly soluble complexes **6** and **7** as polymeric disodium and dipotassium species, respectively (Figure 3). In **6**, one Na fully occupies the fluorenyl‐carbene pocket, while the other interacts with one of the six‐membered rings of the tethered fluorenyl and a neighbouring fluorenyl group. The pocket of **7** is occupied with a single K and the second K interacts with the flanking Dipp aryl ring and the fluorenyl ring on a neighbouring unit. This creates a progression in which, as the size of the cation increases, the more it favours the single occupation of the fluorenyl‐carbene pocket. The Na−carbene distance of 2.578(3) Å closely matches previously reported distances.5d,5g, 6a The K−carbene bond length of 3.011(5) Å also fits within the range of previously reported K–carbene complexes,5a,5b,5g, 6a, 7c, 10a whereas the angle of 23.3° between K and the NCN plane (Table 1) is similar to those in previous structures. The angle between the NCN plane and the metal also fits the trend previously mentioned: as the metal cation increases in size, so does the distortion from planarity. The yaw angle is less affected by the change in metal cation. The M−N bond length between the metal bound to the NHC and the amide increases with the metal ion size and remains longer than the M−N distance to the other metal ion. A variety of coordination modes to fluorenide anions have been previously observed. With sodium, interactions are dominated by η^5^‐ and slipped η^5^‐geometries,^22^ whereas potassium interactions are more varied.^23^

**Figure 3:**
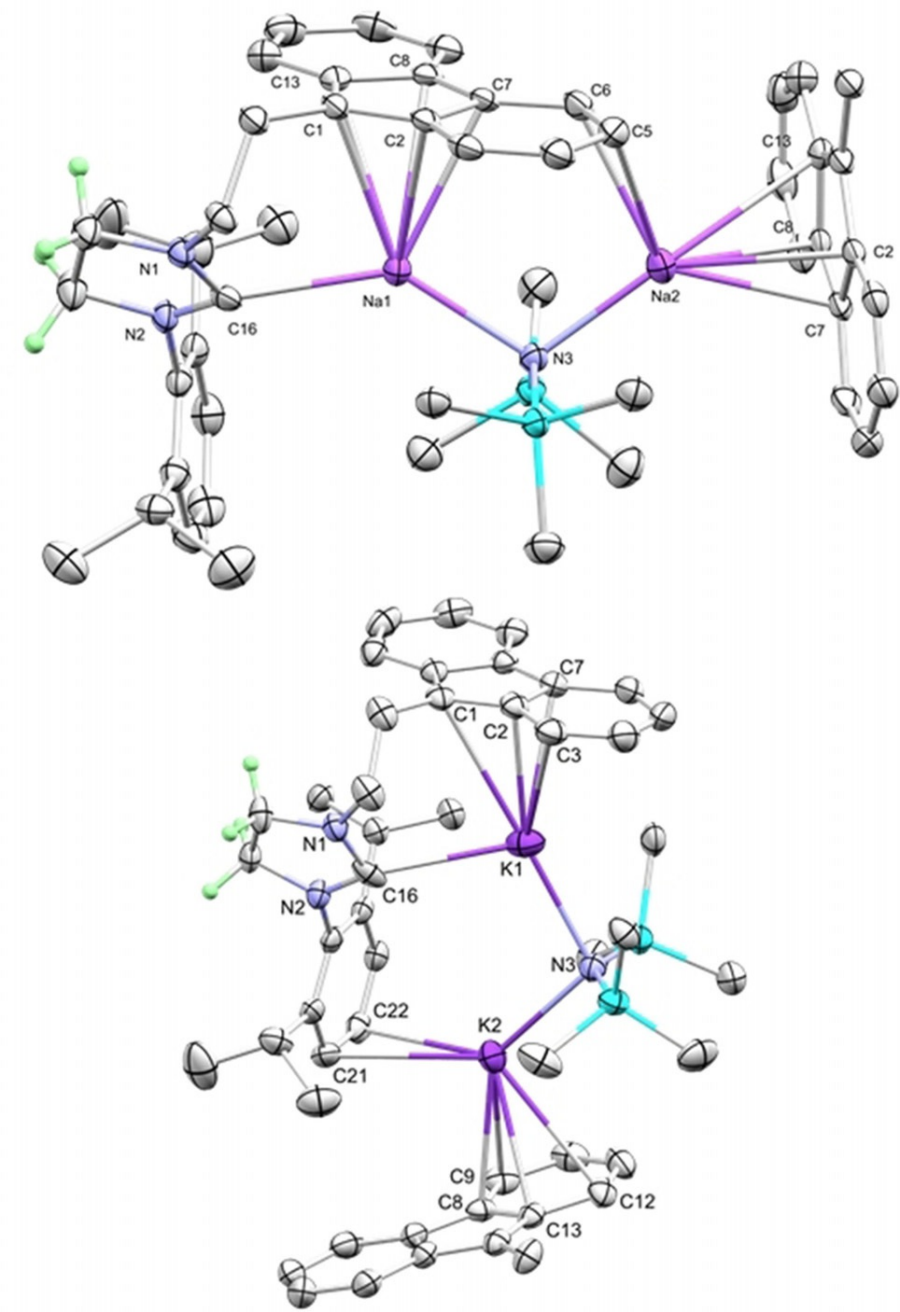
Molecular structures of **6** and **7** (thermal ellipsoids at 50 %). All H atoms have been removed for clarity, except on the saturated NHC backbone. The fluorenyl ring system of additional molecules have been displayed to demonstrate their extended nature.

**Table 1:** Selected structural parameters for **3**, **6** and **7**.

	**3**	**6**	**7**
M−NHC [Å]	2.109(3)	2.578(3)	3.011(5)
M‐NHC pitch angle [°]^[a]^	11.1	18.7	23.3
M‐NHC yaw angle [°]^[b]^	7.1	7.2	8.9
M1−N [Å]	1.949(3)	2.424(3)	2.795(3)
M2−N [Å]	1.967(3)	2.361(2)	2.727(3)

[a] Deviation from planarity of M to the NCN plane. [b] Deviation from linearity of the M‐NHC angle.

Preliminary studies have shown that **3** acts as a useful ligand‐transfer reagent in the reaction with [Rh(CO)_2_Cl]_2_, in which coordination of both the NHC and fluorenide donors to a Rh(CO) fragment is observed. The presence of LiN(SiMe_3_)_2_ does not interfere with this reaction, and work in this direction is currently ongoing.

In summary, synergic combinations of alkali‐metal reagents have been used to access tethered saturated‐NHC complexes forming lithium, sodium and potassium homobimetallic complexes. The lithium complexes feature a bridging amide between the two Li atoms within the fluorenyl‐NHC pocket and are monomeric in nature. This contrasts with the sodium and potassium complexes that are polymeric because the fluorenyl‐NHC pocket is filled by only one metal cation leading to the other metal cation forming interactions between molecules. The structure of the homobimetallic species LiPh/LiN(SiMe_3_)_2_ revealed a Li_2_(μ^2^‐X)_2_ core forming a polymeric structure through additional Li–η^3^‐Ph interactions.^24^

## Conflict of interest

The authors declare no conflict of interest.

## Supporting information

As a service to our authors and readers, this journal provides supporting information supplied by the authors. Such materials are peer reviewed and may be re‐organized for online delivery, but are not copy‐edited or typeset. Technical support issues arising from supporting information (other than missing files) should be addressed to the authors.

Supplementary
