# Supplementary material for: Synergic Deprotonation Generates Alkali‐Metal Salts of Tethered Fluorenide‐NHC Ligands Co‐Complexed to Alkali‐Metal Amides
Source: Chemistry. 2019 Feb 19;25(15):3766–9. doi: 10.1002/chem.201806278 (PMC6492165; doi:10.1002/chem.201806278)
Supplement: Supplementary file 1 — Supplementary [file CHEM-25-3766-s001.pdf]

CHEMISTRY
A European Journal

Supporting Information

Synergic Deprotonation Generates Alkali-Metal Salts of Tethered Fluorenide-NHC Ligands Co-Complexed to Alkali-Metal Amides

Kieren J. Evans and Stephen M. Mansell*[a]

chem_201806278_sm_miscellaneous_information.pdf

Contents

1. General Remarks 2

2. Synthesis of Compounds 2

2.1 Synthesis of Flu-Dipp Imidazolinium BF_4 **1** 2

2.2 Synthesis of Flu-Dipp Spirocycle **2** 3

2.3 Isolation of LiPh/LiN($SiMe_3$)$_2$ adduct **5** 4

2.4 Flu-Dipp NHC DiLi [μ-N($SiMe_3$)$_2$] **3** 4

2.5 Flu-Dipp NHC DiLi (μ-TMP) **4** 5

2.6 Flu-Dipp NHC DiNa [μ-N($SiMe_3$)$_2$] **6** 5

2.7 Flu-Dipp NHC DiK [μ-N($SiMe_3$)$_2$] **7** 6

3. NMR Spectra 7

3.1 Stacked plot NMR spectra of reaction mixtures 13

4. Mass Spectra 19

5. Crystallographic Data 20

1. General Remarks

All reactions requiring inert conditions were performed using standard Schlenk-line techniques or in a glovebox under an atmosphere of dinitrogen. Dry toluene, THF and MeCN were obtained from a solvent purification system (MBraun SP-300) and stored over 4 Å molecular sieves prior to use. Petroleum ether (40-60°C) was dried over sodium wire, distilled and stored over 4 Å molecular sieves prior to use. Diethyl ether was dried over sodium/benzophenone, distilled and stored over 4 Å molecular sieves prior to use. C_6D_6 and C_6H_6 was dried over molten potassium, distilled and stored in the glovebox prior to use. $(C_{13}H_9)C_2H_4N(H)C_2H_4N(H)(C_{12}H_{15})$ was synthesised as previously reported.[1] Lithium bis(trimethylsilyl)amide (LiHMDS) was purchased from Sigma Aldrich and used as received. Phenyllithium and lithium 2,2,6,6-tetramethylpiperidide were synthesised accordingly to literature procedures and stored in the glovebox.[2]

NMR spectra were obtained on either a Bruker AV 400, AVIII 400 or AVIIIHD spectrometer. ^{1}H NMR was recorded at 400 MHz and spectra referenced to the residual solvent peak (7.16 for C_6D_6 and 1.94 for CD_3CN). ^{13}C{^{1}H} NMR spectra was recorded at 101 MHz and the spectra referenced to the internal solvent peak (128.06 ppm for C_6D_6 and 118.26 for CD_3CN). ^{7}Li NMR were recorded at 155 MHz and referenced to an external standard of LiCl in D_2O. ^{11}B NMR were recorded at 128 MHz. ^{19}F NMR spectra was recorded at 377 MHz. ^{29}Si{^{1}H} NMR were recorded at 75.9 MHz using an INEPT pulse sequence and referenced to an external standard of Me_4Si.

Melting points were recorded on a Stuart SMP-10 instrument and are quoted with the solvent of crystallisation.

Mass spectrometry was conducted at the UK ESPRC Mass Spectrometry Facility at Swansea University using the techniques stated.

Elemental analysis were performed by Dr Brian Hutton (Heriot-Watt University, non air-sensitive) and Mr Stephen Boyer (London Metropolitan University, air sensitive). For the homobimetallic compounds, we experienced great difficulties in obtaining satisfactory elemental analyses. Compound **3** (with LiN'', pKa = 26) required multiple analyses to get satisfactory data with widely differing results despite high purities of the samples (as judged by multinuclear NMR spectroscopy). Despite many attempts, we could not achieve satisfactory analyses for the other compounds. The much higher pKa values for LiPh (43) and LiTMP (37) are likely to reflect their higher sensitivities.

2. Synthesis of Compounds

2.1 Synthesis of Flu-Dipp Imidazolinium BF_4 **1**

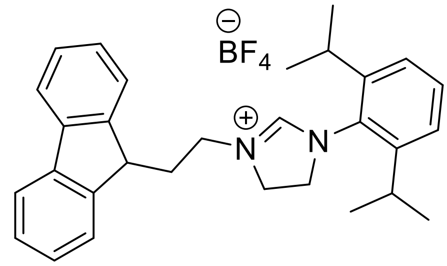

In a Schlenk flask, $(C_{13}H_9)C_2H_4N(H)C_2H_4N(H)(C_{12}H_{15})$ (5.348 g, 12.96 mmol), NH_4BF_4 (2.10 g, 18.3 mmol, 1.5 eq.) and triethyl orthoformate (20 cm^3) were combined in toluene (15 cm^3) with a few drops of formic acid. The reaction mixture was heated at 100°C overnight. Upon cooling, the volatiles were removed under reduced pressure and the residue was extracted with CH_2Cl_2 (15 cm^3) to remove excess

NH_4BF_4. The filtrate was concentrated and diethyl ether added to precipitate the crude product. The crude was purified with column chromatography (SiO_2, MeCN/CH_2Cl_2, 3:17→1:3 v/v). Recrystallisation from CH_2Cl_2/diethyl ether afforded the product as a colourless solid (4.376 g, 8.57 mmol, 66%). Single crystals suitable for X-ray diffraction were obtained from slow cooling a hot C_6H_6 solution.

R_f: 0.24 (MeCN/CH_2Cl_2, 1:4); MP (CH_2Cl_2/diethyl ether): 166-168°C; ^{1}H NMR (CD_3CN, 400 MHz, 298 K): δ 7.86-7.88 (m, 2 H, Ar), 7.80 (s, 1 H, NC*H*N), 7.64-7.67 (m, 2H, Ar), 7.38-7.48 (m, 5H, Ar), 7.31 (d, J = 7.7Hz, 2H, Ar), 4.20 (d, J = 5.6Hz, 1H, Flu-H), 3.99 (m, 4H, overlapping NCH_2CH_2N), 3.30-3.34 (m, 2H, NC*H2*CH2Flu), 2.87 [app. sept, J = 6.8Hz, $CH(CH_3)_2$], 2.51-2.56 (m, 2H, NCH_2CH_2Flu), 1.23 [d, J = 6.8Hz, 6H, $CH(CH_3)_2$], 1.17 [d, J = 6.8Hz, 6H, $CH(CH_3)_2$]; ^{13}C NMR (CD_3CN, 101 MHz, 298 K): δ 158.6 (Imid CH), 147.9, 146.6, 142.0, 132.1, 131.1, 128.7, 128.4, 125.9, 125.6, 121.2, 54.3 (NCH_2CH_2N), 50.1 (NCH_2CH_2N), 46.3 (N*C*H_2CH_2, 45.8 (Flu-H), 30.8 (CH_2CH_2Flu), 29.2 ($CHMe_2$), 24.9 (CH_3), 24.4 (CH_3); ^{19}F (CD_3CN, 377 MHz, 298 K) δ -151.8 (s); ^{11}B (CD_3CN, 128 MHz, 298 K) δ -1.17; IR (thin film)/cm^{-1} 3068(w), 2966(w), 1642(s), 1449(m), 1270(w), 1055(s), 740(m); HRMS(ESI$^+$) 423.2788 $[M-BF_4]^+$, $C_{30}H_{35}N_2$ requires 423.2795; $C_{30}H_{35}N_2BF_4$: calcd. C 70.59, H 6.91, N 5.49; found C 70.59, H 6.92, N 5.20.

2.2 Synthesis of Flu-Dipp Spirocycle **2**

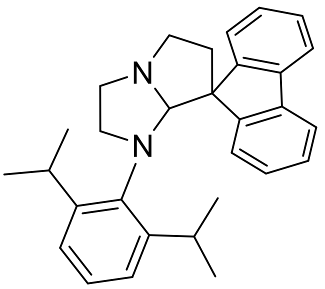

To a stirred suspension of **1** (1.2 g, 2.24 mmol, 1.1 eq.) in diethyl ether (20 cm^3), *n*-BuLi (1.4 ml, 1.512 M, 2.12 mmol, 1 eq.) was added at -78°C. The reaction was allowed to warm to room temperature. A red solution initially formed which slowly faded to a colourless, clear solution. After 2 hours, the solvent was removed under reduced pressure and the resultant white residue dried thoroughly under vacuum. The residue was extracted with pet ether (3 x 10 cm^3) and filtered. Removal of the solvent gave the product as a pure, colourless solid (714 mg, 1.63 mmol, 80%). Crystals suitable for X-ray diffraction were obtained from a concentrated pet ether solution.

MP (pet ether): 123-125°C; ^{1}H NMR (C_6D_6, 400 MHz, 298 K): δ 7.66-7.64 (m, 1H, Ar), 7.47 (dq, J = 5.5, 0.6 Hz, 1H, Ar), 7.38-7.36 (m, 1H, Ar), 7.31-7.26 (m, 2H, Ar), 7.17-7.15 (m, 1H, overlap with C_6H_6), 6.99-6.85 (m, 4H, Ar), 6.73 (dd, J = 5.5, 1.8Hz, 1H, Ar), 5.45 (s, 1H, NC*H*N), 3.63-3.52 (m, 3H), 3.47 (ddd, J = 10.1, 7.8, 2.1 Hz, 1H), 3.11-2.98 (m, 3H), 2.48-2.37 (m, 2H), 1.95 (ddd, J = 13.1, 6.5, 2.2Hz, 1H), 1.40 (d, J = 6.7Hz, CH3), 1.18 (d, J = 6.8Hz, CH_3), 0.80 (d, J = 6.9Hz, CH_3), 0.68 (d, J = 6.9Hz, CH_3); ^{13}C NMR (C_6D_6, 101 MHz, 298 K): δ 150.1 (Dipp-Ar), 149.8, 147.1 (Dipp-Ar), 145.0, 141.9, 140.9, 127.8, 127.1, 127.0, 127.0, 126.9. 125.3, 123.1, 122.3, 120.3, 119.8, 98.8 [CH, N*C*H(Flu)], 62.1 (4°), 57.6 (NCH_2CH_2N), 55.0 (NCH_2CH_2N), 53.7 (N*C*H_2CH_2Flu), 40.2 (CH_2Flu), 27.2 (CH), 26.7 (CH), 26.1 (CH_3), 25.2 (CH_3), 24.7 (CH_3), 23.2 (CH_3); HRMS(ASAP$^+$) 423.2800 $[M+H]^+$, $C_{30}H_{35}N_2$ requires 423.2795; $C_{30}H_{34}N_2$: calcd. C 85.26, H 8.11, N 6.63; found C 85.08, H 8.20, N 6.69.

2.3 Isolation of LiPh/LiN(SiMe$_3$)$_2$ adduct **5**

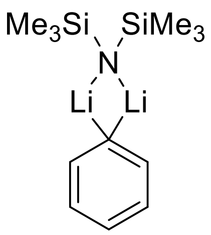

LiPh (336 mg, 4.00 mmol) and LiN(SiMe$_3$)$_2$ (670 mg, 4.00 mmol) were combined in toluene (10 cm^3) and mixed for 5 mins. The solution was then concentrated under reduced pressure and left overnight at 20°C for the product to crystallise out. The supernatant solution was filtered away and the solid washed with cold pet ether (2 x 10 cm^3). The solid was dried under vacuum, obtaining the product as an off-white microcrystalline solid (510 mg, 2.03 mmol, 51%). Single crystals suitable for X-ray diffraction were obtained from concentrated C_6H_6 solutions.

^{1}H NMR (C_6D_6, 400 MHz, 298 K): δ 8.06 (dd, J = 7.4, 1.6Hz, 2H, *ortho*-ArH), 7.13 (t, J = 7.4Hz, 2H, *meta*-ArH overlaps with residual solvent peak for C_6D_6), 6.98-6.94 (m, 1H, *para*-ArH), 0.09 (s, 18H, SiMe$_3$); ^{13}C NMR (C_6D_6, 101MHz, 298 K) δ 181.2 (Ar-Li), 144.9 (*ortho*-ArH), 128.1 (*meta*-ArH, overlaps with C_6D_6 peak), 126.9 (*para*-ArH), 5.6 (SiMe$_3$); ^{7}Li NMR (C_6D_6, 155.5 MHz, 298 K) δ -0.55 (s); ^{29}Si NMR (C_6D_6, 79.5 MHz, 298 K) δ -10.92 (s).

2.4 Flu-Dipp NHC DiLi [μ-N(SiMe$_3$)$_2$] **3**

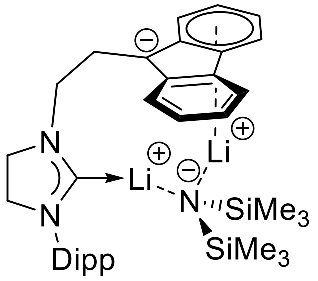

2 (400 mg, 0.946 mmol), LiHMDS (174.3 mg, 1.041 mmol) and LiPh (87.5 mg, 1.041 mmol) were combined in toluene (5 cm^3) and the sample was heated for 2 days at 80°C. The reaction was allowed to cool and all of the volatiles were removed under reduced pressure. Toluene (1 cm^3) and pet ether (20 cm^3) were added and the mixture was vigorously stirred for 5 hr. After standing for 30 mins, the resultant red precipitate was isolated by filtration, washed with pet ether (5 cm^3) and dried under vacuum affording the product as a red solid (495 mg, 0.831 mmol, 88%). Crystals suitable for X-ray diffraction were obtained from a concentrated C_6H_6 solution.

^{1}H NMR (C_6D_6, 400 MHz, 298 K): δ 8.26 (ddd, 2H, *J* = 7.8, 1.1, 0.8 Hz), 7.52 (d, 2H, *J* = 8.2 Hz), 7.32 (ddd, 2H, *J* = 8.1, 6.7, 1.2 Hz) 7.01 (dd, 1H, *J* = 8.3, 7.2 Hz), 6.90-6.85 (m, 4H, overlap), 3.63-3.60 (m, 2H, NC*H*$_2$CH$_2$Flu), 3.25-3.17 (m, 4H, NCH$_2$CH$_2$N), 3.16-3.13 (m, 2H, NCH$_2$C*H*$_2$Flu), 2.81 (app. sept, 2H, *J* = 6.8 Hz), 1.10 (d, 6H, *J* = 6.9 Hz), 0.99 (d, 6H, *J* = 6.8 Hz), -0.30 (s, 18H, SiMe$_3$); ^{13}C NMR (C_6D_6, 101 MHz, 298 K): δ 147.3 (Ar *C*-CH(CH$_3$)$_2$), 137.1 (Ar *C*-N), 136.2 (Ar C 1 to 9-Flu), 128.8 (Ar CH *para* to N), 124.3 (Ar CH *meta* to N), 121.6 (Ar C 6 to 9-Flu), 121.4 (Ar CH 4 to 9-Flu), 120.8 (Ar CH 5 to 9-Flu), 114.8 (Ar CH 2 to 9-Flu), 109.1 (Ar CH 3 to 9-Flu), 90.8 (9-Flu), 54.1 (NCH$_2$CH$_2$N), 52.8 (N*C*H$_2$CH$_2$Flu), 49.4 (NCH$_2$CH$_2$N), 28.1 (*C*H(CH$_3$)$_2$), 25.4 (NCH$_2$*C*H$_2$Flu), 25.3 (CH$_3$), 25.0 (CH$_3$), 5.1 (SiMe$_3$); ^{7}Li NMR (C_6D_6,

155.5 MHz, 298 K): δ -0.86, -5.68; ^{29}Si NMR (C_6D_6, 79.5 MHz, 298 K): δ -10.00; $C_{36}H_{51}Li_2N_3Si_2$: calcd. C 72.56, H 8.63, N 7.05; found C 72.46, H 8.67, N 6.91.

2.5 Flu-Dipp NHC DiLi (μ-TMP) **4**

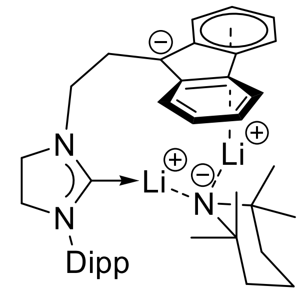

2 (400 mg, 0.946 mmol), LiTMP (139.2 mg, 0.946 mmol) and LiPh (79.5 mg, 0.946 mmol) were combined in toluene (5 cm^3) and the sample was heated for 1 day at 80°C. The reaction was allowed to cool and all of the volatiles were removed under reduced pressure. Toluene (1 cm^3) and pet ether (20 cm^3) were added and the mixture was vigorously stirred for 5 hr. After standing for 30 mins, the resultant red precipitate was isolated by filtration, washed with pet ether (5 cm^3) and dried under vacuum affording a red solid (349 mg, 0.605 mmol, 64%). Recrystallisation from toluene at -78°C afforded crystalline material which was isolated by filtration and dried under vacuum to give the product as a red-orange solid (93 mg, 0.16 mmol, 17%). Crystals suitable for X-ray diffraction were obtained from a concentrated C_6H_6 solution.

^{1}H NMR (C_6D_6, 400 MHz, 298 K): δ 8.73 (d, 2H, *J* = 7.8 Hz), 7.54 (d, 2H, *J* = 8.2 Hz), 7.32 (app. t, 2H, *J* = 6.9 Hz) 7.01 (t, 1H, *J* = 7.2 Hz), 6.94-6.89 (m, 4H, overlap), 3.57-3.54 (m, 6H, overlap), 3.27-3.21 (m, 2H, NCH_2CH_2Flu), 2.71 (app. sept, 2H, *J* = 6.8 Hz), 1.48-1.45 (m, 1H), 1.34-1.29 (m, 2H), 1.19-1.14 (m, 3H), 1.07 (d, 6H, *J* = 6.9 Hz), 0.99 (d, 6H, *J* = 6.8 Hz), 0.77 (s, 6H, Me), 0.31 (s, 6H, Me); ^{7}Li NMR (C_6D_6, 155.5 MHz, 298 K): δ 0.09, -5.40.

Small scale syntheses of Na and K compounds

2.6 Flu-Dipp NHC DiNa [μ-N($SiMe_3$)$_2$] **6**

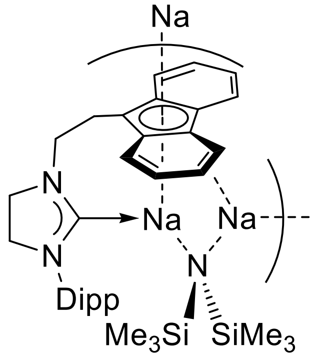

2 (50 mg, 0.118 mmol), NaHMDS (21.7 mg, 0.118 mmol) and $NaCH_2Ph$ (13.5 mg, 0.118 mmol) were combined in toluene (5 cm^3). The reaction mixture was heated for 30 mins at 80°C and then left to cool overnight. The mixture was filtered at 0°C and the remaining solid dried under vacuum to obtain the product as an orange solid (14 mg, 0.0214 mmol, 18%). Crystals suitable for X-ray diffraction were

obtained from a NMR scale reaction (0.047 mmol) in C_6D_6 after transferring the sample to a vial and leaving to crystallise for 1 week.

Compound **6** (and **7**) showed very poor solubility in benzene and toluene precluding NMR spectroscopic analysis.

2.7 Flu-Dipp NHC DiK [μ-N(SiMe$_3$)$_2$] **7**

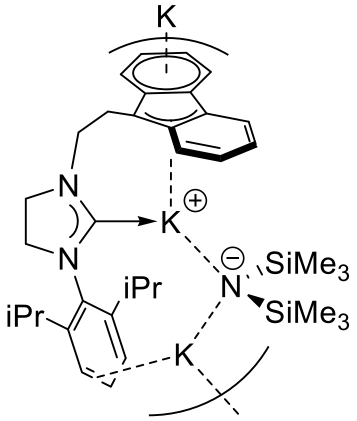

2 (50 mg, 0.118 mmol), KHMDS (23.6 mg, 0.118 mmol) and KCH_2Ph (15.4 mg, 0.118 mmol) were combined in toluene (5 cm^3). The reaction mixture was heated for 30 mins at 80°C and then left to cool overnight. The mixture was filtered at 0°C and the remaining solid dried under vacuum to obtain the product as an orange solid (40 mg, 0.060 mmol, 51%). Crystals suitable for X-ray diffraction were obtained from a NMR scale reaction (0.047 mmol) in C_6D_6 after transferring the sample to a vial and leaving to crystallise for 3 weeks.

[1] M. Rosello-Merino, S. M. Mansell, *Dalton Trans.* **2016**, *45*, 6282-6293.

[2] a) A. J. Roberts, A. R. Kennedy, R. McLellan, S. D. Robertson, E. Hevia, *Eur. J. Inorg. Chem.* **2016**, *2016*, 4752-4760; b) M. F. Lappert, M. J. Slade, A. Singh, J. L. Atwood, R. D. Rogers, R. Shakir, *J. Am. Chem. Soc.* **1983**, *105*, 302-304.

3. NMR Spectra

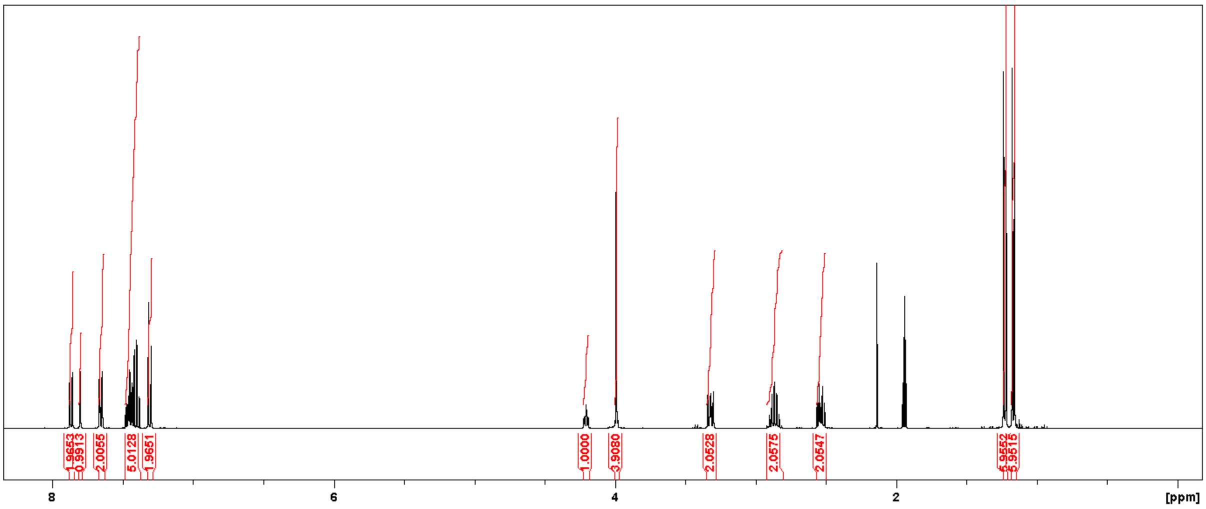

Figure S1: ^{1}H NMR spectrum (400 MHz, CD_3CN, 298 K) for **1**

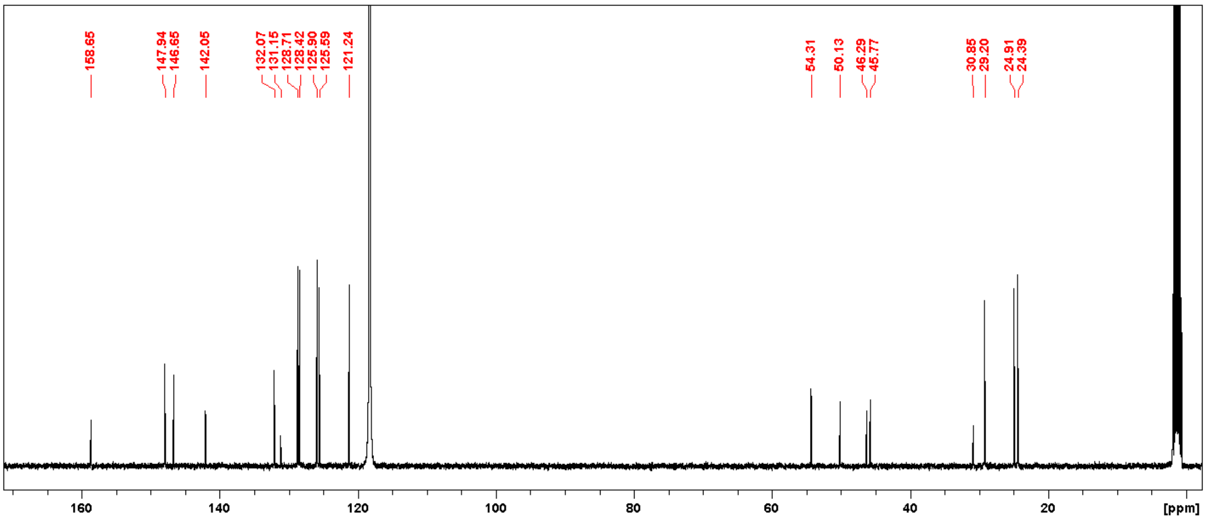

Figure S2: ^{13}C {^{1}H} NMR spectrum (101 MHz, CD_3CN, 298 K) for **1**

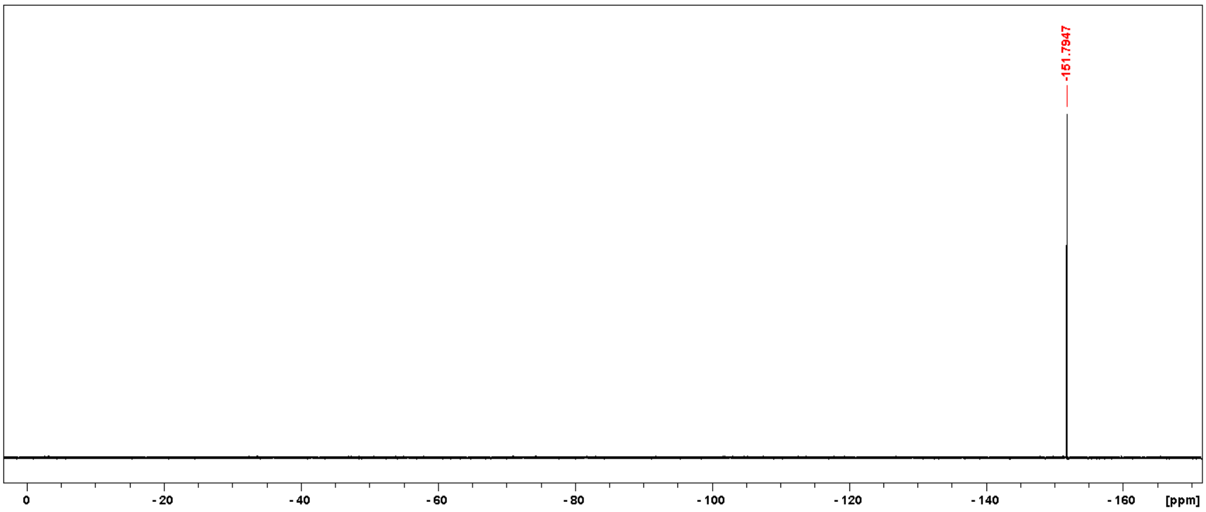

Figure S3: ^{19}F NMR spectrum (277 MHz, CD_3CN, 298 K) for **1**

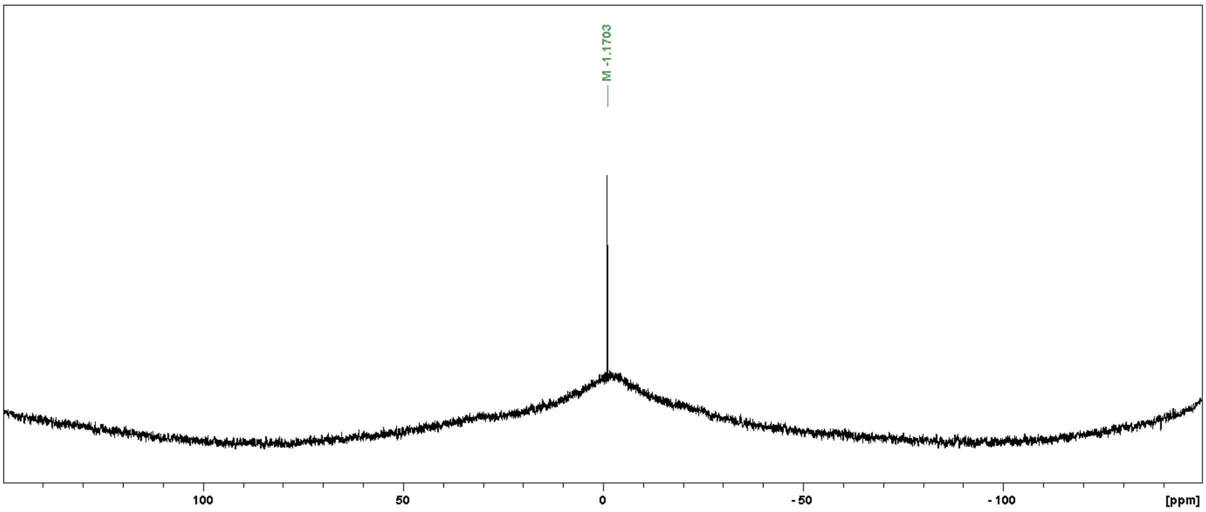

Figure S4: ^{11}B NMR spectrum (128 MHz, CD_3CN, 298 K) for **1**

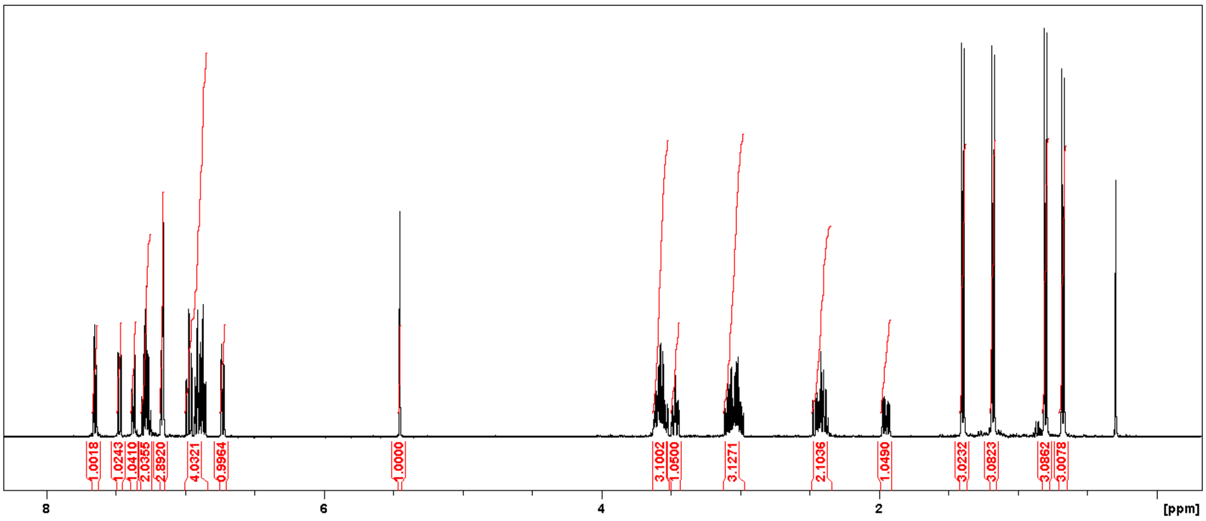

Figure S5: ^{1}H NMR spectrum (400 MHz, C_6D_6, 298 K) for **2**

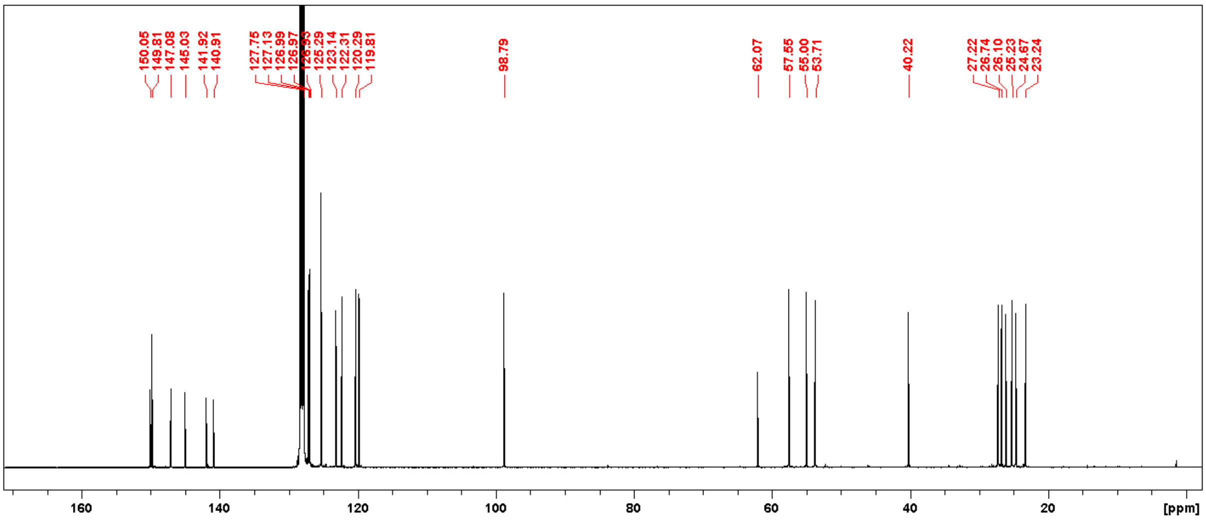

Figure S6: ^{13}C {^{1}H} NMR spectrum (101 MHz, C_6D_6, 298 K) for **2**

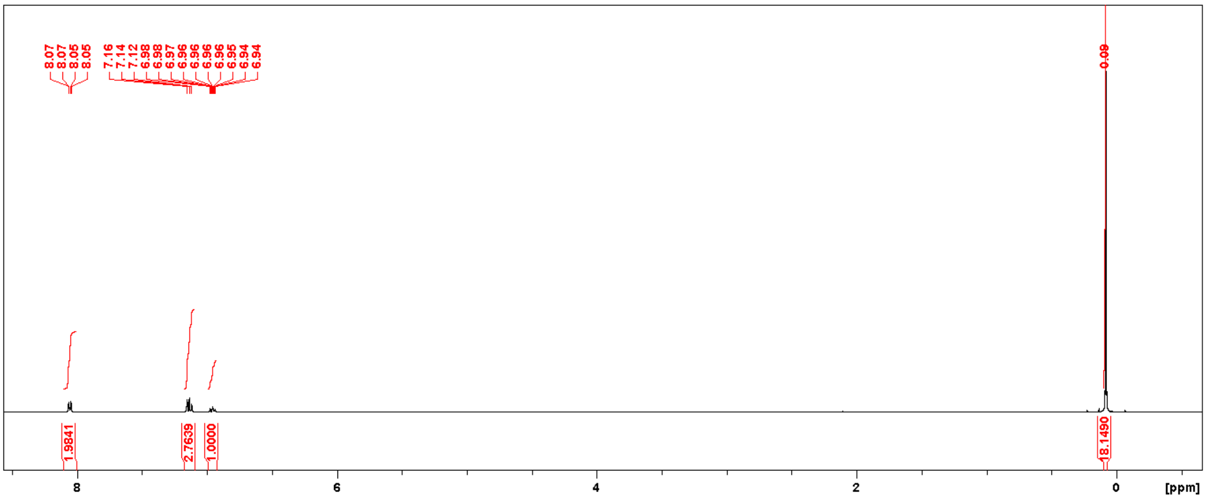

Figure S7: ^{1}H NMR spectrum (400 MHz, C_6D_6, 298 K) for **5**

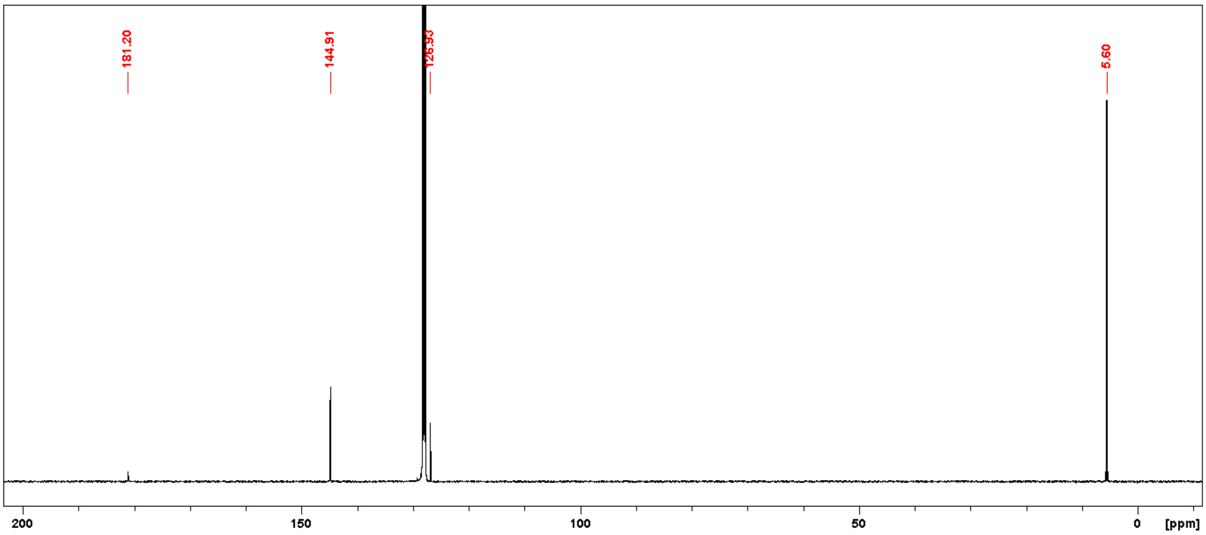

Figure S8: ^{13}C {^{1}H} NMR spectrum (101 MHz, C_6D_6, 298 K) for **5**

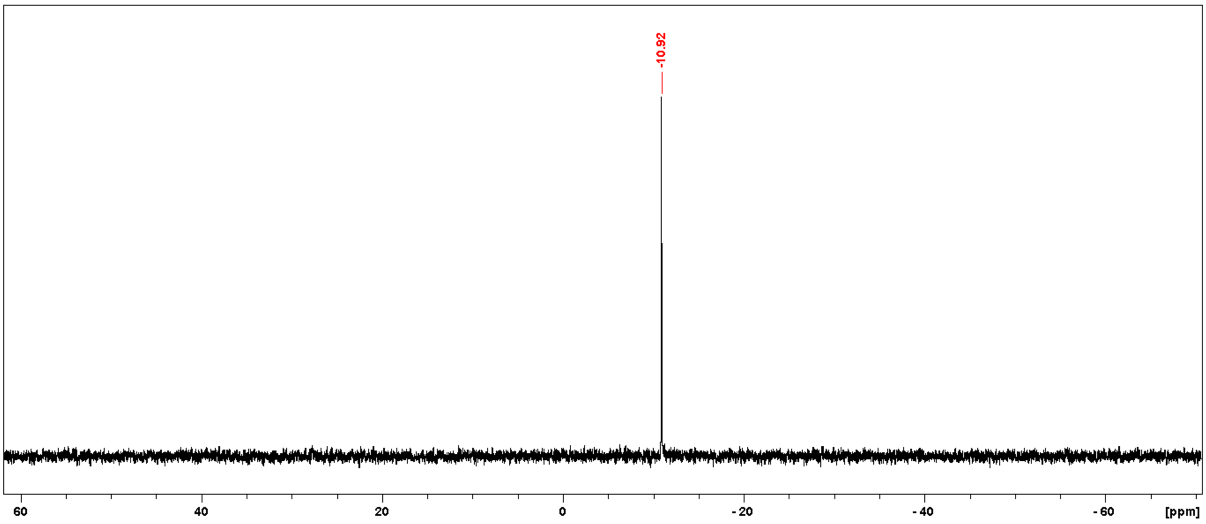

Figure S9: ^{29}Si NMR spectrum (79.5 MHz, C_6D_6, 298 K) for **5**

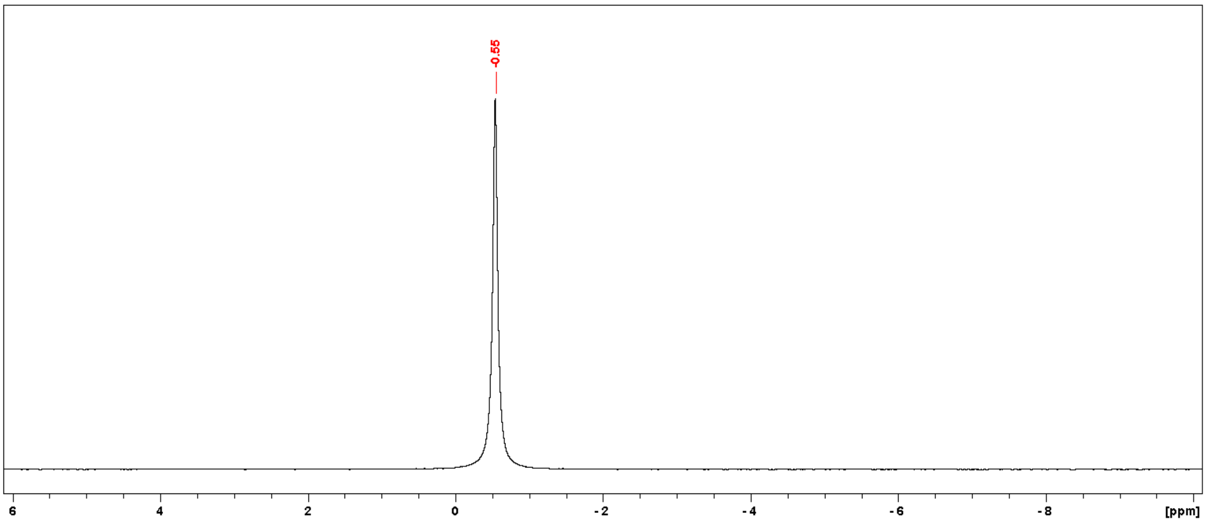

Figure S10: ^{7}Li NMR spectrum (155.5 MHz, C_6D_6, 298 K) for **5**

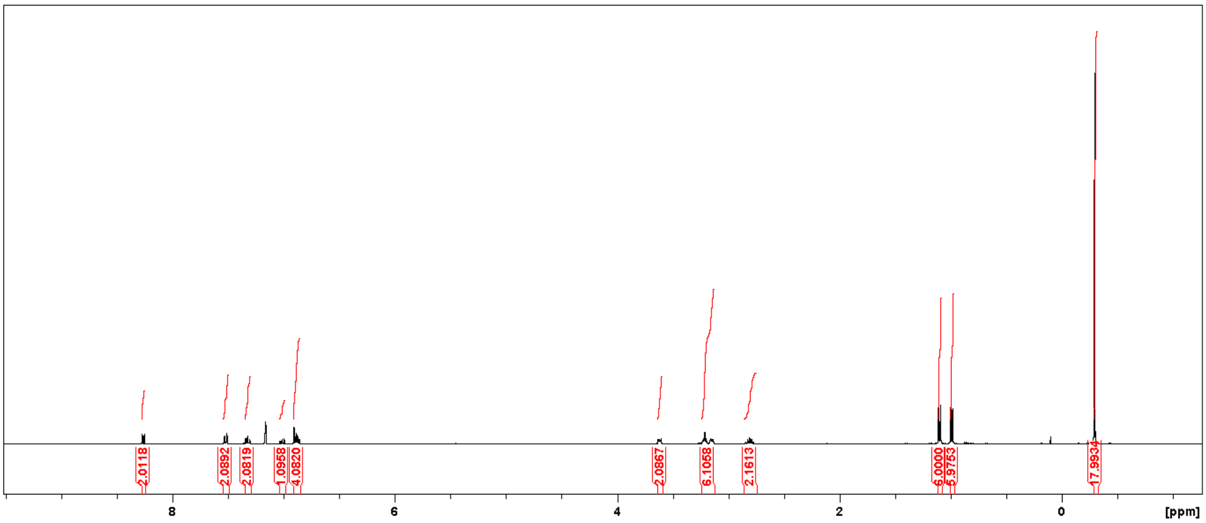

Figure S11: ^{1}H NMR spectrum (400 MHz, C_6D_6, 298 K) for **3**

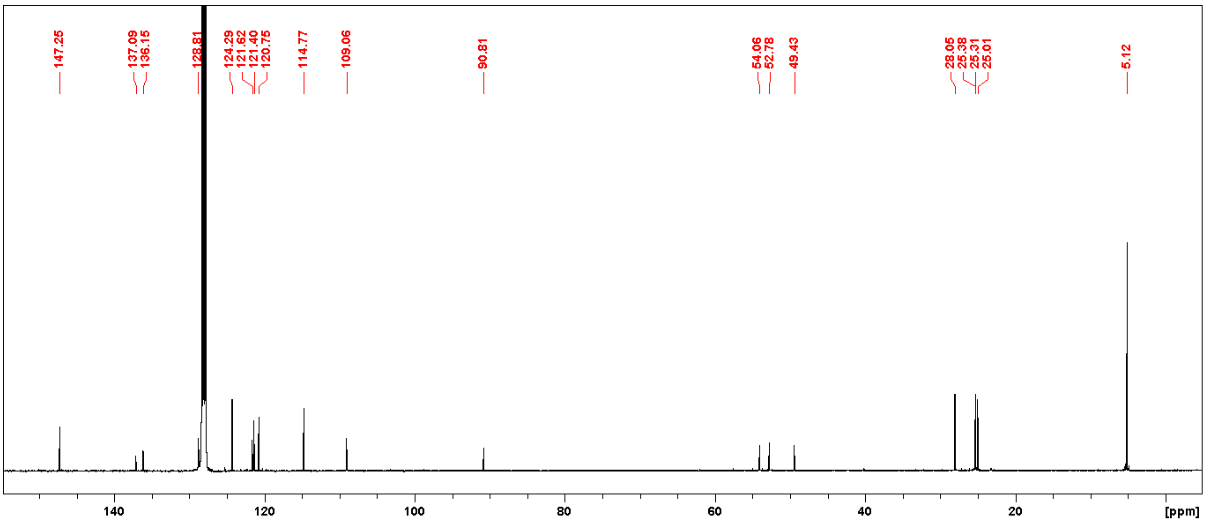

Figure S12: ^{13}C {^{1}H} NMR spectrum (101 MHz, C_6D_6, 298 K) for **3**

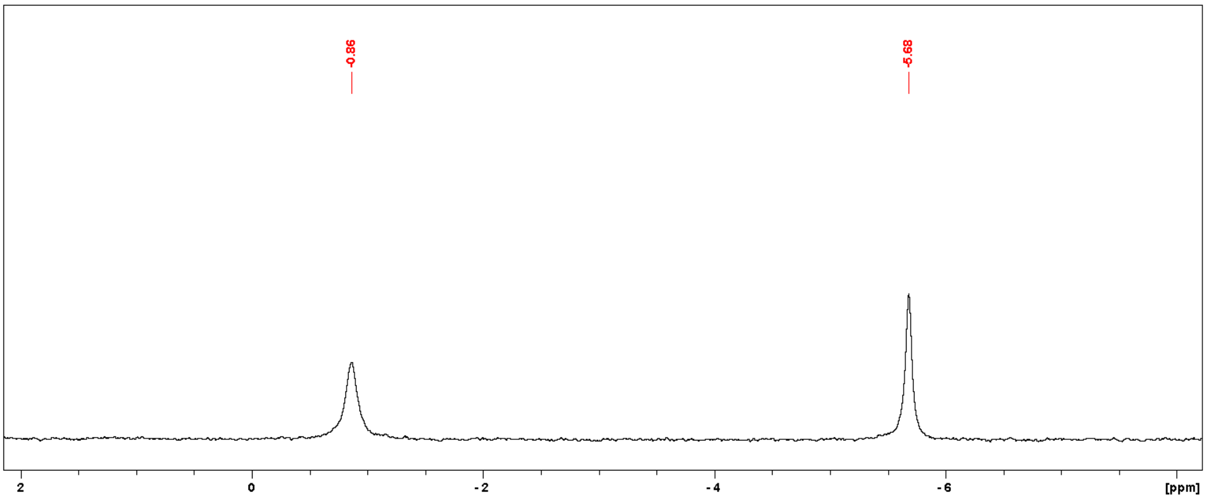

Figure S13: ^{7}Li NMR spectrum (155.5 MHz, C_6D_6, 298 K) for **3**

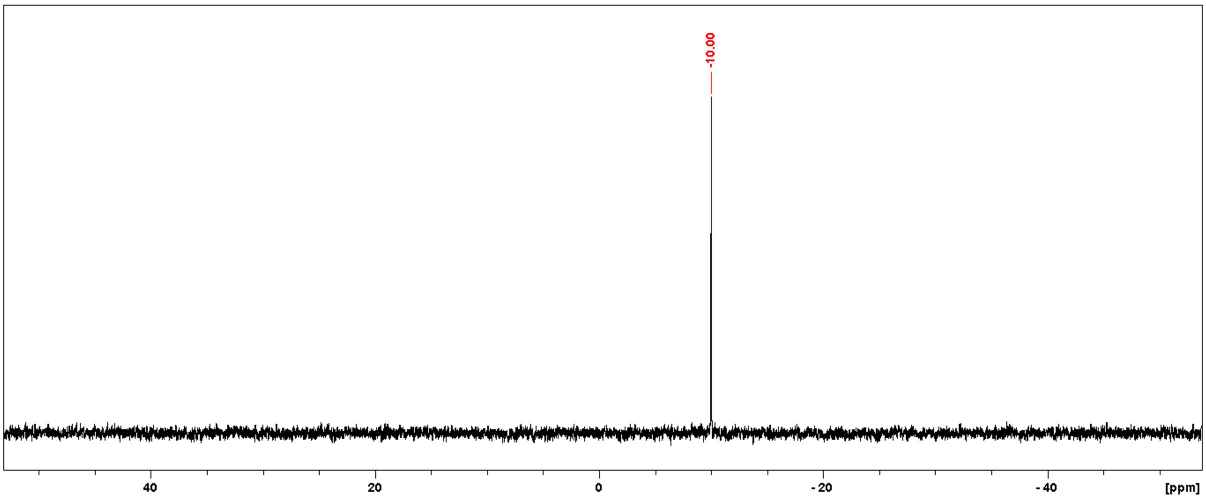

Figure S14: ^{29}Si NMR spectrum (79.5 MHz, C_6D_6, 298 K) for **3**

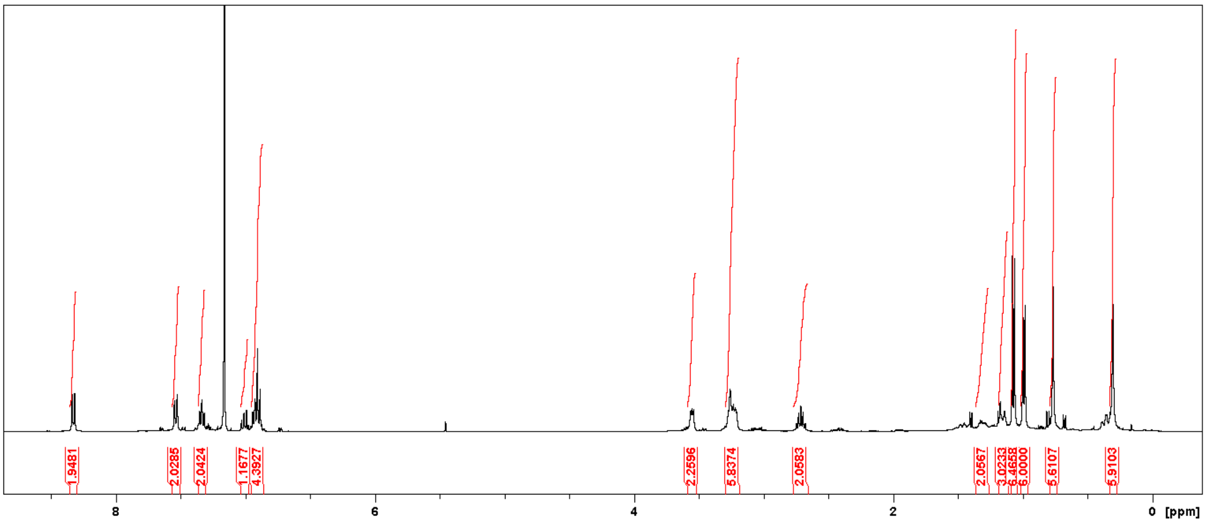

Figure S15: ^{1}H NMR spectrum (400 MHz, C_6D_6, 298 K) for **4** from an NMR scale reaction after 3 hrs at 80°C.

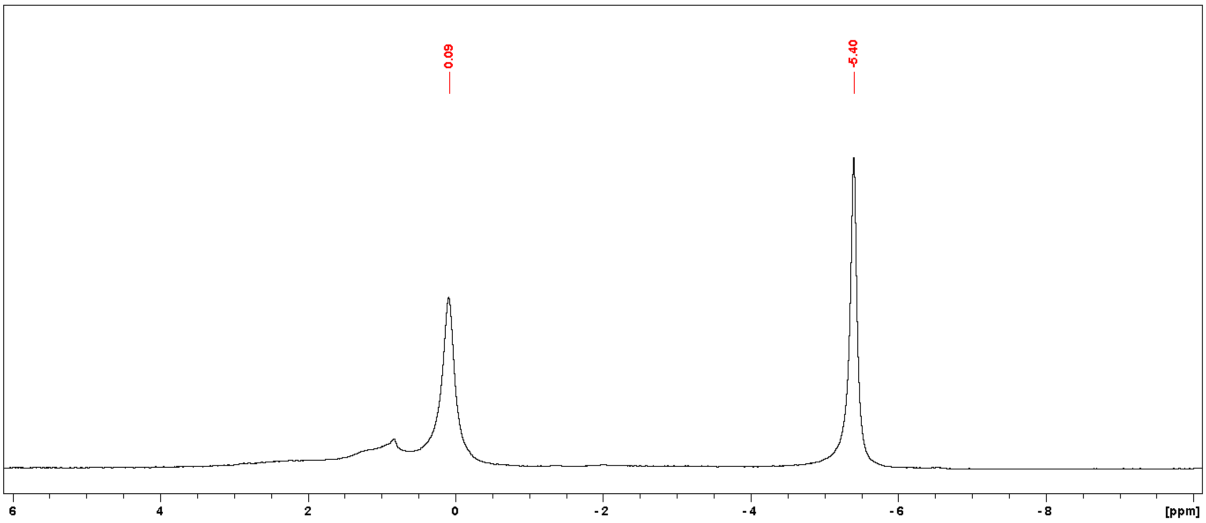

Figure S16: ^{7}Li NMR spectrum (155 MHz, C_6D_6, 298 K) for **4** from a NMR scale reaction after 3hrs at 80°C.

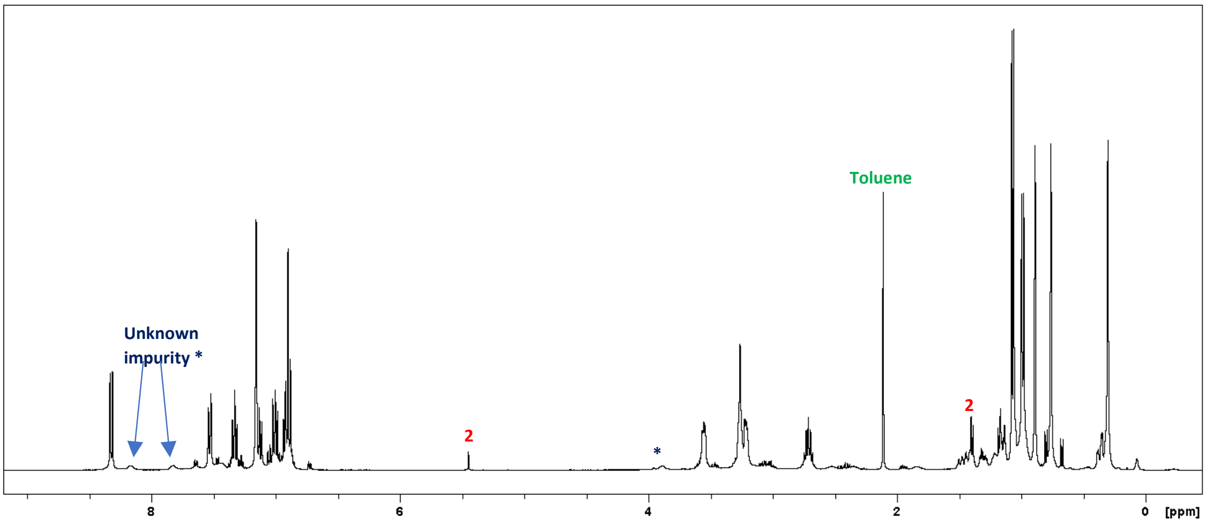

Figure S17: ^{1}H NMR spectrum (400 MHz, C6D6, 298K) for **4** from a preparative scale reaction after recrystallisation from toluene/pet ether and subsequent recrystallisation at -78°C in toluene. The presence of **2** could be from either incomplete removal or degradation due to exposure to moisture, while toluene appears to be trapped in the crystalline solid despite drying at 4.4 x 10^{-3} mbar.

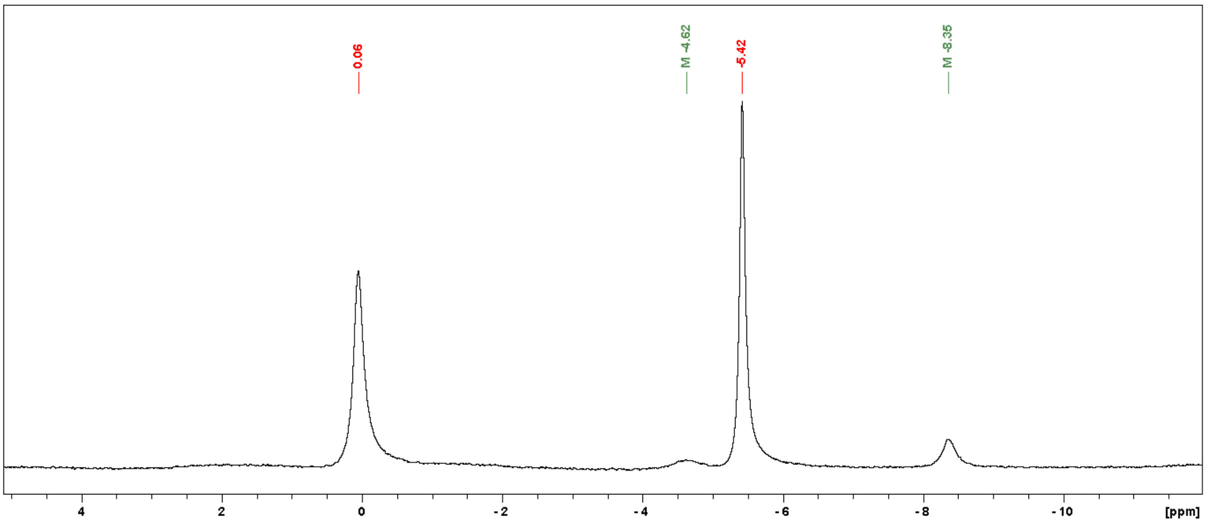

Figure S18: ^{7}Li NMR spectrum (155 MHz, C_6D_6, 298 K) for **4** a preparative scale reaction after recrystallisation from toluene/pet ether and subsequent recrystallisation at -78°C in toluene.

3.1 Stacked plot NMR spectra of reaction mixtures

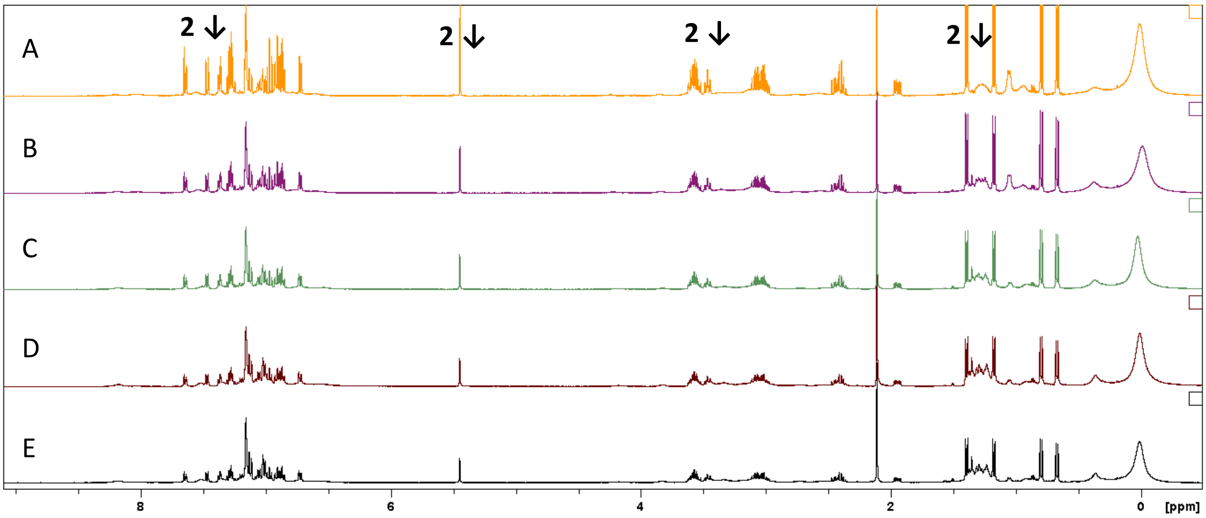

Figure S19: Stacked plot of ^{1}H NMR spectra for NMR scale reaction of **2** with $NaCH_2Ph$/NaHMDS at room temperature (A: 1 hr, B: 3 hr, C: 5.5 hr, D: 18 hr, E: 24 hr). A decrease in signal intensity for **2** was observed and there are only weak and broad signals indicating the poor solubility of **6**.

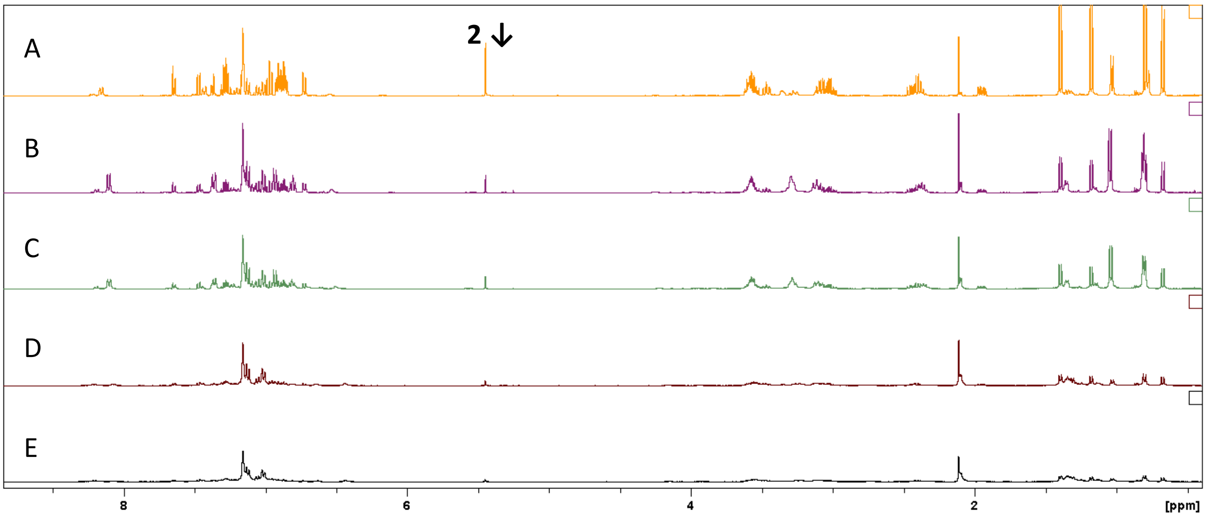

Figure S20: Stacked plot of ^{1}H NMR spectra for NMR scale reaction of **2** with KCH_2Ph/KHMDS at room temperature (HMDS region omitted for clarity, A: 1 hr, B: 3 hr, C: 5.5 hr, D: 18 hr, E: 24 hr). D and E clearly indicate the poor solubility of **7**.

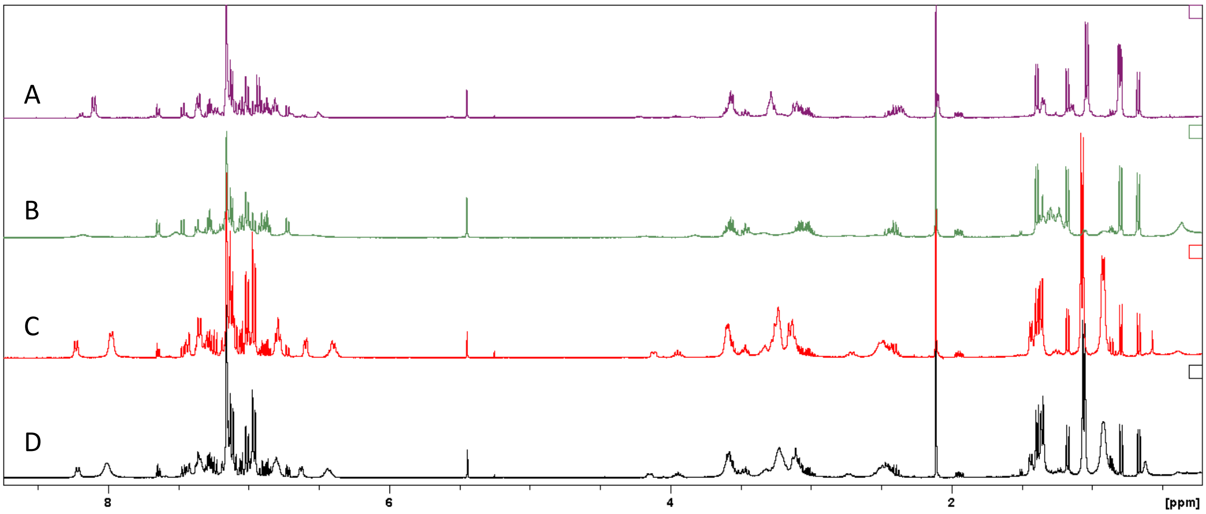

Figure S21: Stacked plot of ^{1}H NMR spectra for NMR scale reaction of **2** with A: KCH_2Ph/KHMDS, B: $NaCH_2Ph$/NaHMDS, C: KCH_2Ph/NaHMDS, D: $NaCH_2Ph$/KHMDS. The spectra for C and D are very similar in nature suggesting the same product(s) are being formed. Crystals grown from the same reactions as D proved to those of **7**, indicating that an exchange must be occurring.

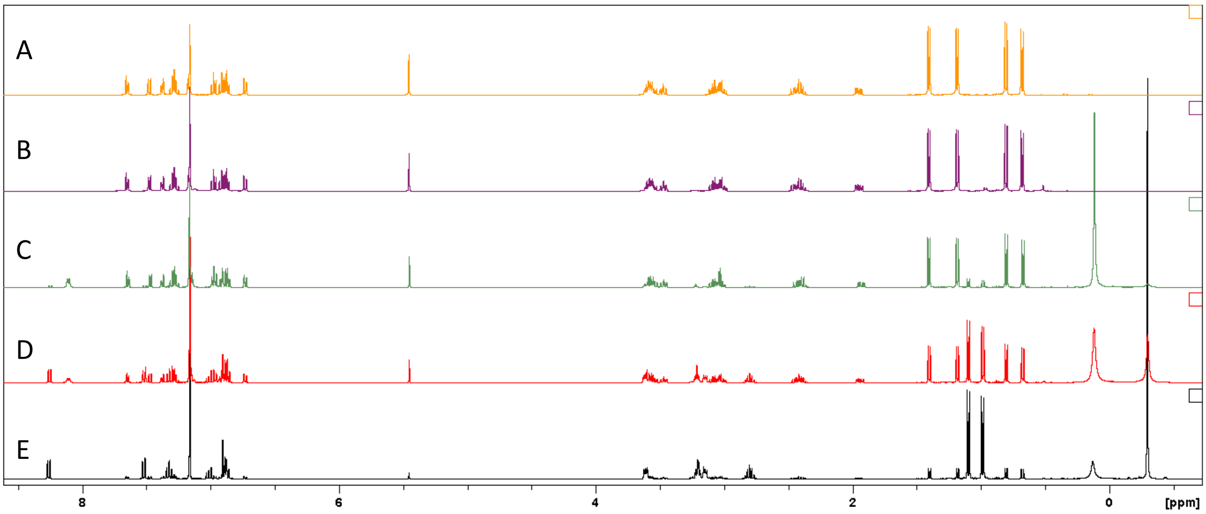

Figure S22: Stacked plot of ^{1}H NMR spectra for NMR scale reaction of **2** with a) LiPh on mixing, b) after 1hr at 80°C, c) on addition of LiHMDS, d) after 1hr at 80°C, and e) after further 3 hrs at 80°C. The conversion to **3** in E is 80% which is typical of reactions of 2 with mixed LiPh/LiHMDS after 4hrs at 80°C.

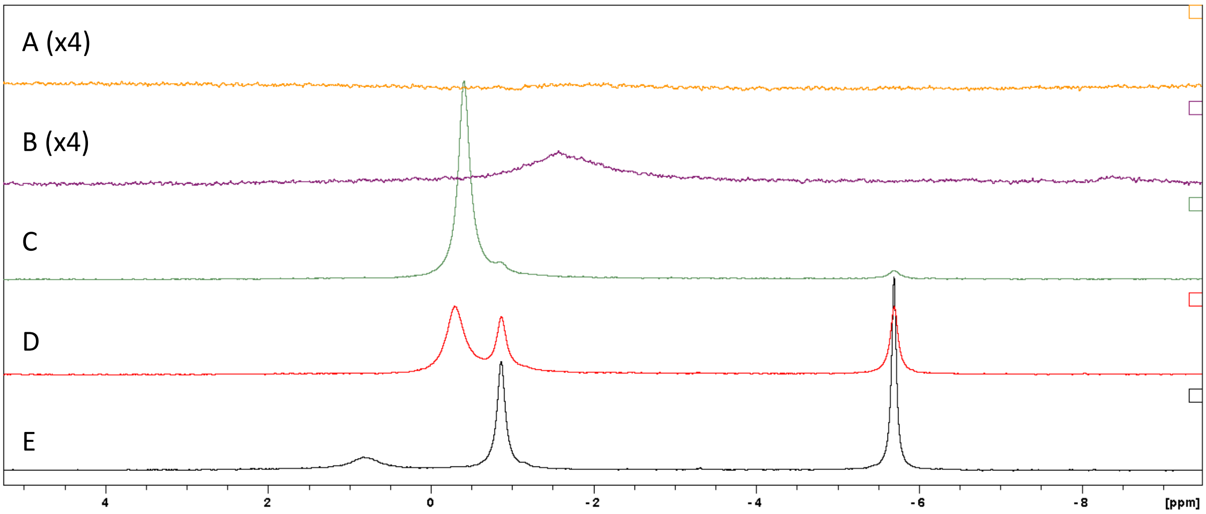

Figure S23: Stacked plot of ^{7}Li NMR spectra for NMR scale reaction of **2** with a) LiPh on mixing, b) after 1hr at 80°C, c) on addition of LiHMDS, d) after 1hr at 80°C, and e) after further 3 hrs at 80°C. Both A and B are shown at 4x the scale of C-E and indicate the poor solution and reactivity of the LiPh with **2** in a non-coordinating solvent i.e. C_6D_6.

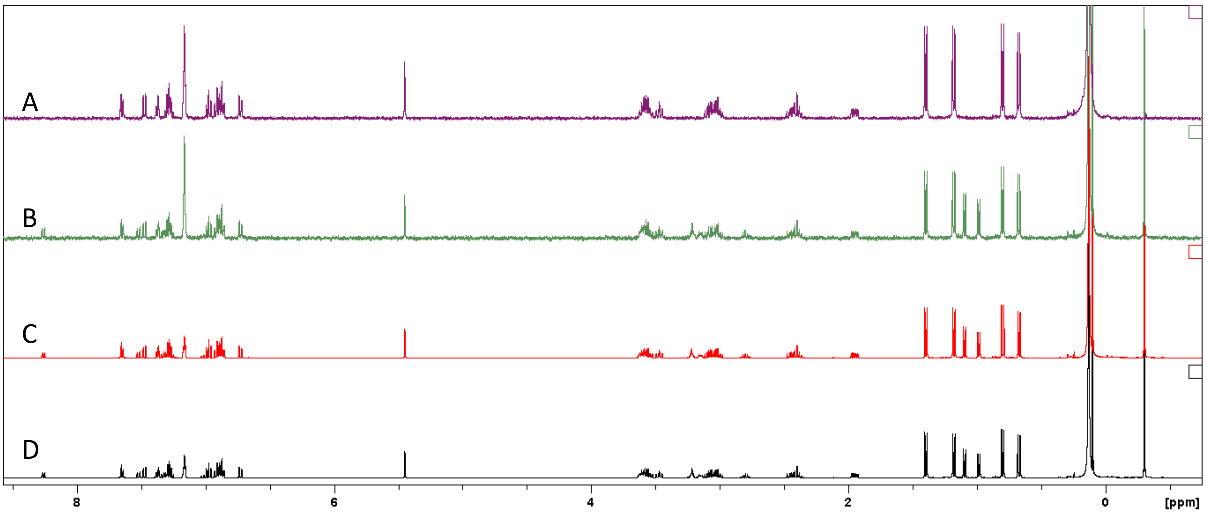

Figure S24: Stacked plot of ^{1}H NMR spectra for NMR scale reaction of **2** with 2 equivalents of LiHMDS a) on mixing, b) after 30min at 80°C, c) extra 1.5hr at 80°C, d) after overnight at 80°C (~16hrs). In C 24% conversion to 3 is observed while in D it has reached 25% conversion, indicating that the reaction has stopped.

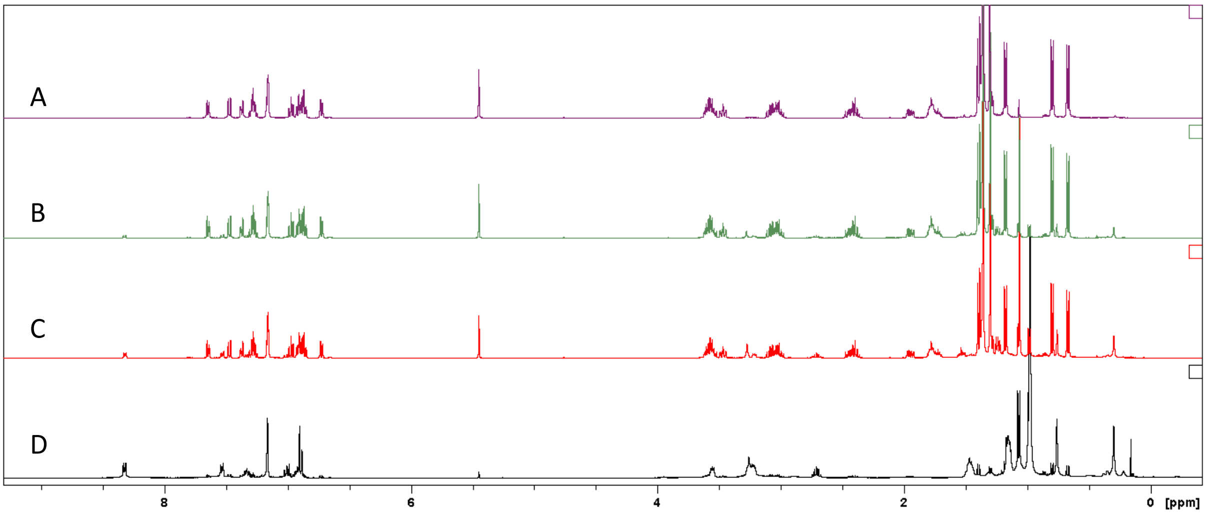

Figure S25: Stacked plot of ^{1}H NMR spectra for NMR scale reaction of **2** with 2 equivalents of LiTMP a) on mixing, b) after 1hr at 80°C, c) extra 1hr at 80°C, d) after overnight at 80°C (~16hrs). In C only 21% conversion to **4** is observed whilst in D it has reached 88% conversion. The reaction of **2** and LiPh/LiTMP achieves >80% within 90mins at 80°C.

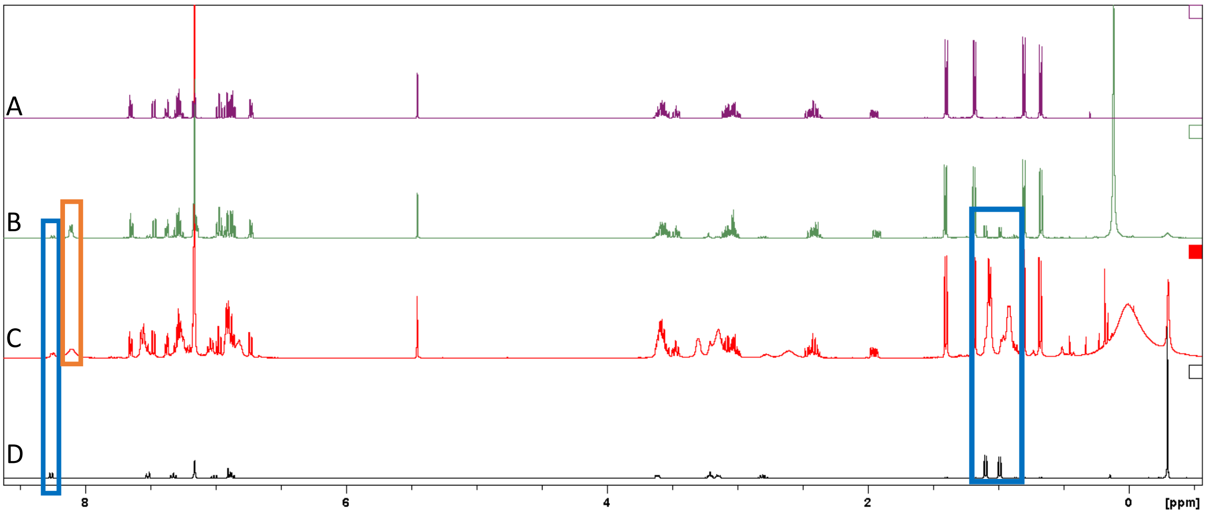

Figure S26: Comparison of ^{1}H NMR A) for spirocyclic **2**, B) **2** with LiPh/LiHMDS after 30mins at 80°C, C) **2** with LiPh/NaHMDS after 1.5hr at 80°C, and D) isolated **3**. The blue highlighted peaks indicated the LiPh/NaHMDS reactions is forming **3**, whilst the orange highlighted ones occur when **2** is mixed with LiPh/LiHMDS suggesting that LiHMDS may be forming in-situ.

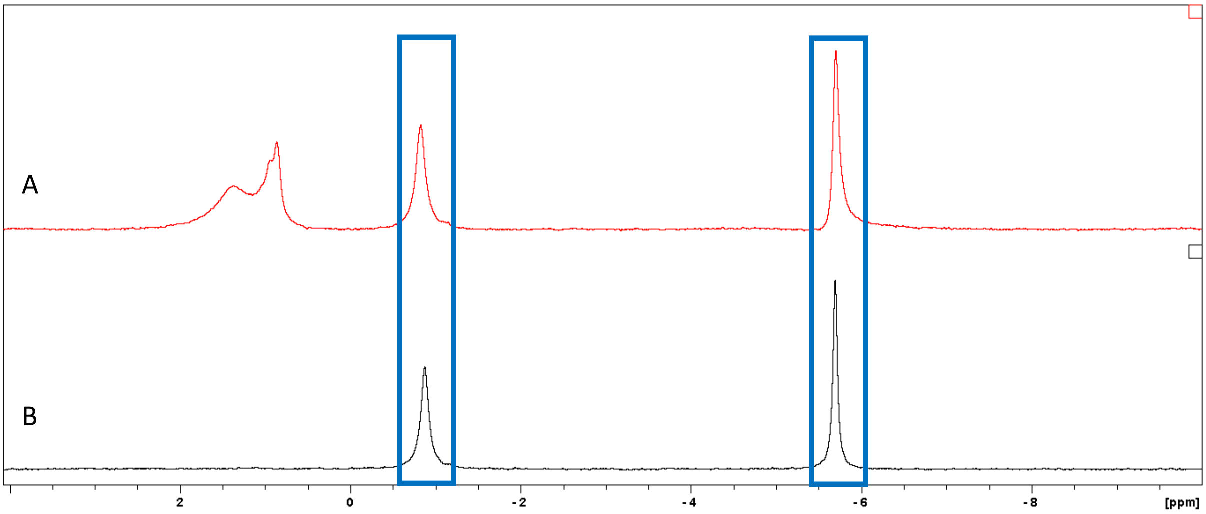

Figure S27: Comparison of ^{7}Li NMR for the reaction of **2** with LiPh/NaHMDS (A) and a sample of **3** (B). From the extreme similarities in the peaks, it would support the hypothesis that the reaction is forming **3**.

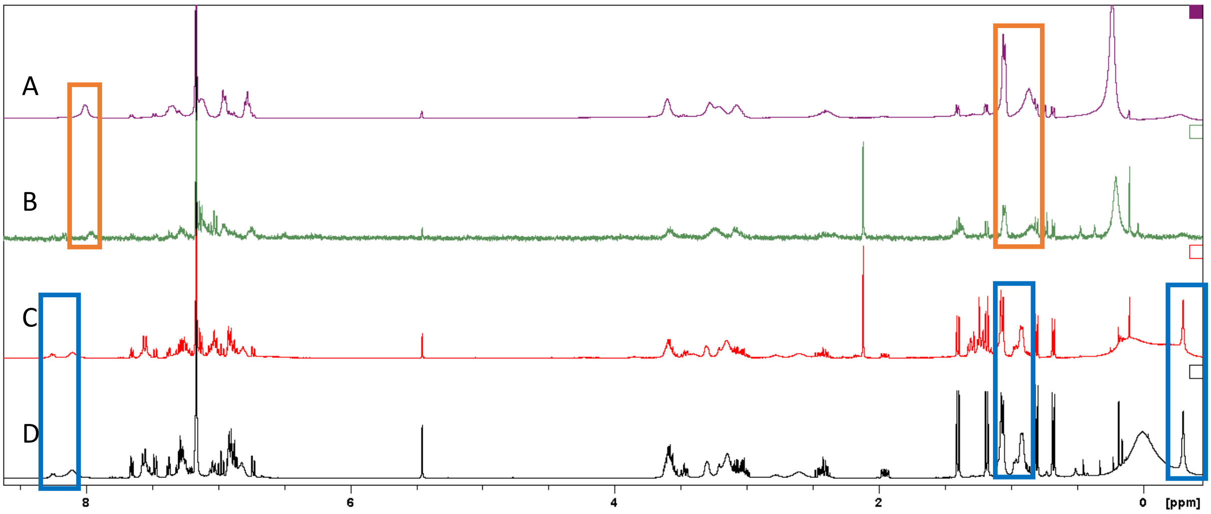

Figure S28: Comparison of ^{1}H NMR for the reactions of **2** with A) LiPh/KHMDS, B) KCH_2Ph/LiHMDS, C) $NaCH_2Ph$/LiHMDS, and D) LiPh/NaHMDS. C and D appear very similar suggesting the same process is happening as in Figures S26 & S27, and that both LiPh/NaHMDS and $NaCH_2Ph$/LiHMDS give the same product **3**. A and B have similarities and presence of toluene in B and the broadness of A could account for any shift differences. The orange highlight species appears quite strong and soluble in A, contrasting with the Li NMR in figure S29 suggesting this peak relates to a species that does not contain Li.

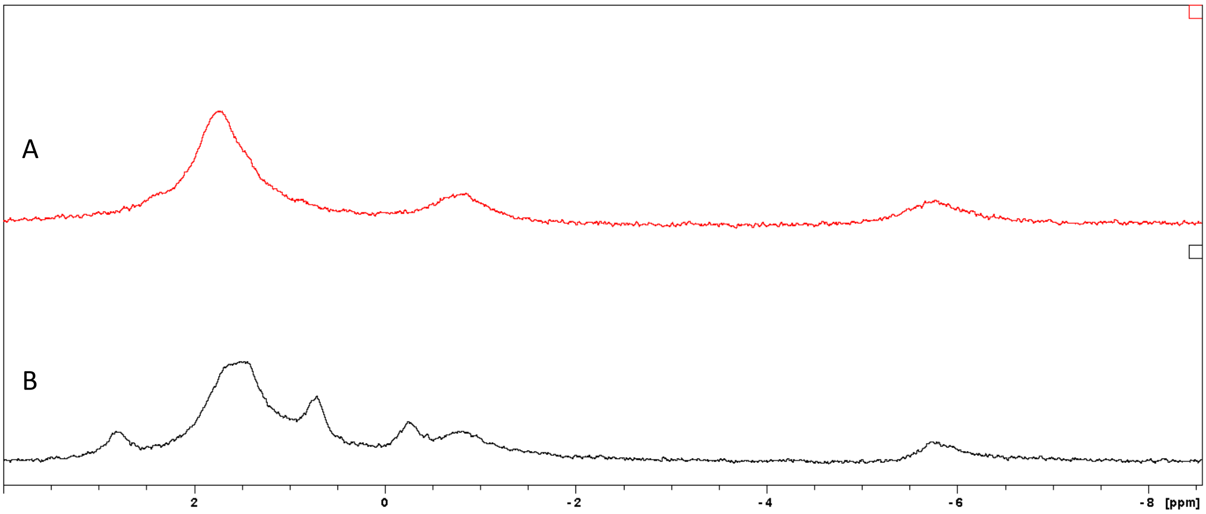

Figure S29: Comparison of ^{7}Li NMR for the reactions of **2** with A) LiPh/KHMDS and B) KCH_2Ph/LiHMDS. As in figure S28, there are similarities though more is going on in B. Both spectra are weak.

4. Mass Spectra

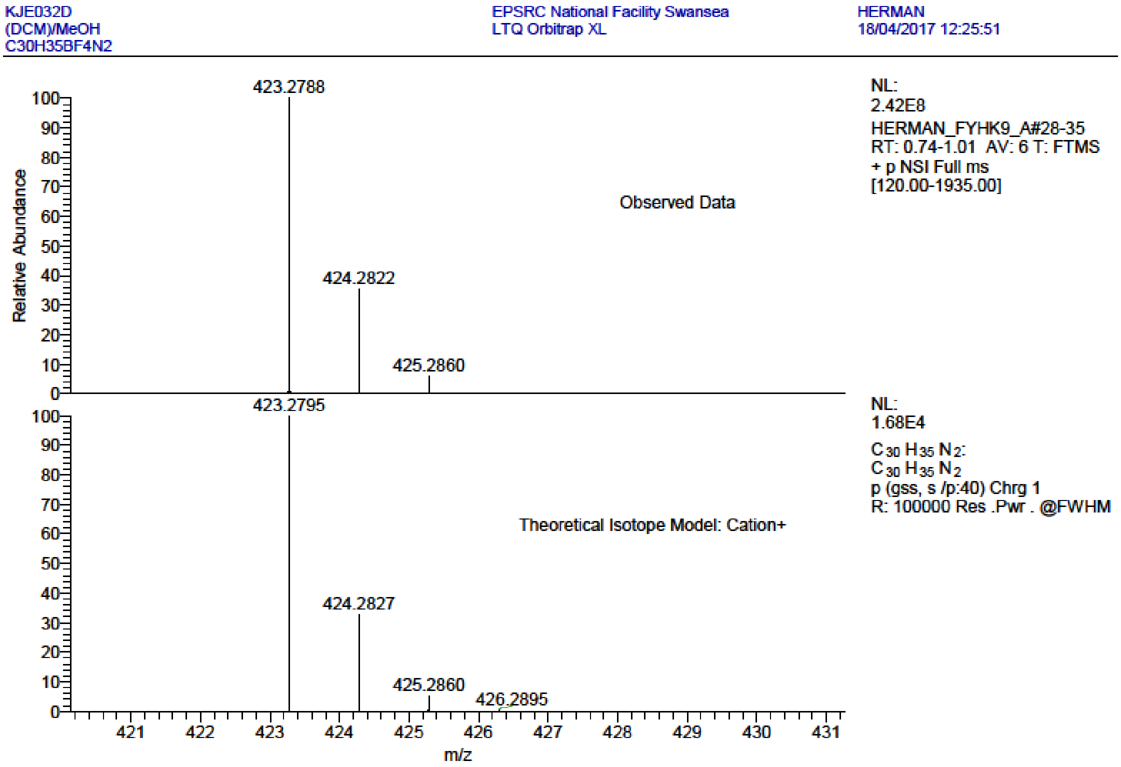

Figure S30: HR(ESI)MS for **1**

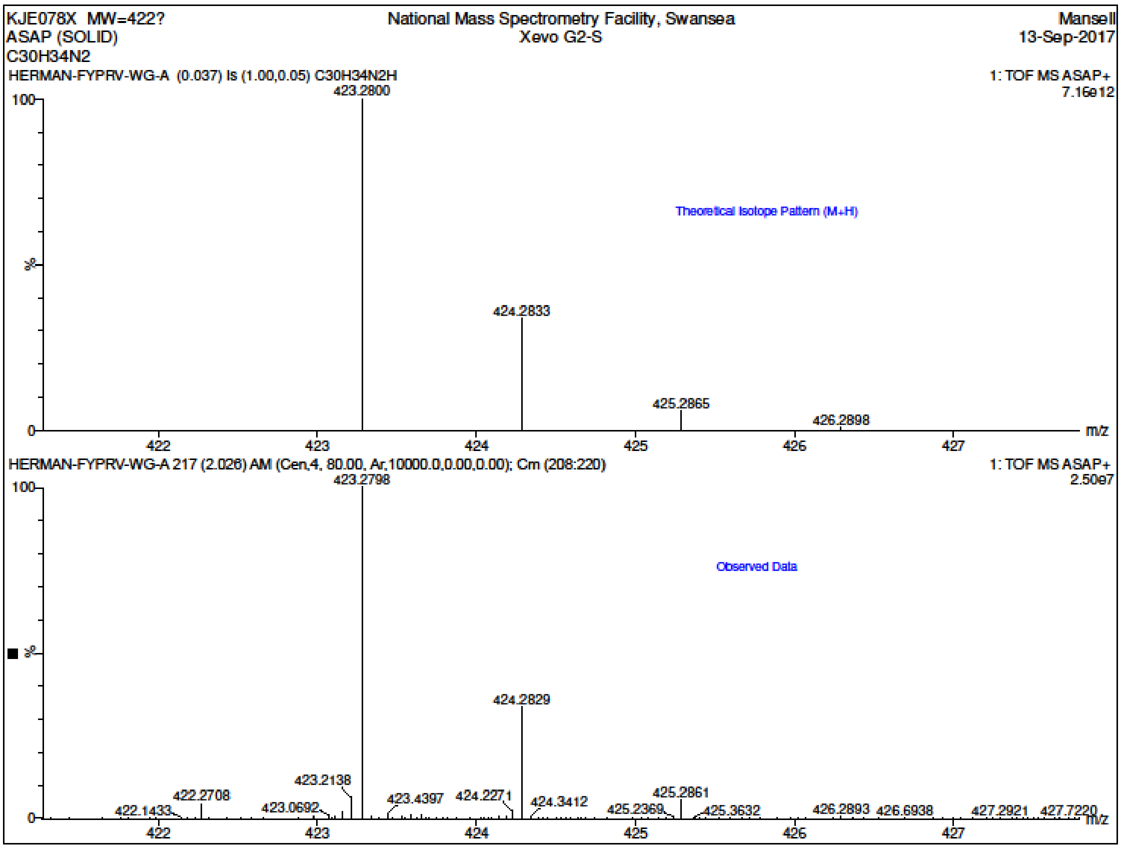

Figure S31: HR(ASAP)MS for **2**

5. Crystallographic Data

Single crystals of the samples were covered in an inert oil and placed under the cold stream of a Bruker X8 APEXII four-circle diffractometer cooled to 100 K (**2** - **6**), or were collected at the National Crystallography service in Southampton on a Rigaku four-circle diffractometer using a rotating Mo anode at 100 K (**1**). Exposures were collected using Mo Kα radiation (λ = 0.71073). Indexing, data collection and absorption correction were performed using the APEXII suite of programs.[1] Structures were solved using direct methods (SHELXT)[2] and refined by full-matrix least-squares (SHELXL)[2] interfaced with the programme OLEX2.[3]

The data for **1** were poor, despite using a powerful rotating anode X-ray source and sensitive detector at the National Crystallography Service, and this combined with twinning led to high R factors. However, the connectivity was still unambiguously determined.

Single crystals of **2** were found to be twinned and the data were refined using an hklf5 refinement as a 2-component twin with the second domain related to the first by rotation of 179.9 degrees about reciprocal axis 0.001 1.000 0.035. Friedel opposites were merged, since anomalous scattering was not significant for a light atom structure from Mo radiation.

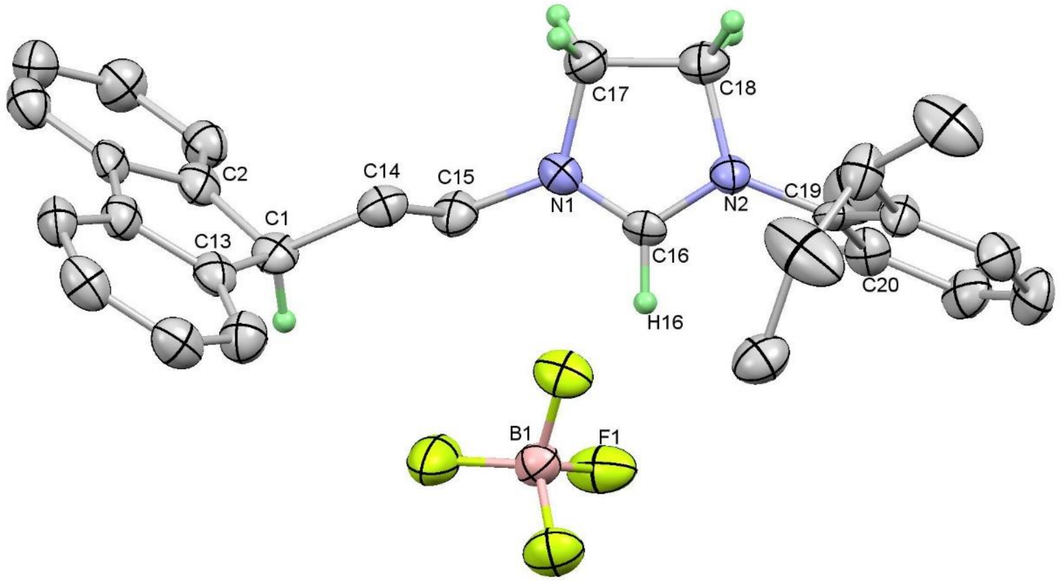

Figure S32. Molecular structure of **1** (ellipsoids at 50% probability). Only selected H atoms are shown for clarity.

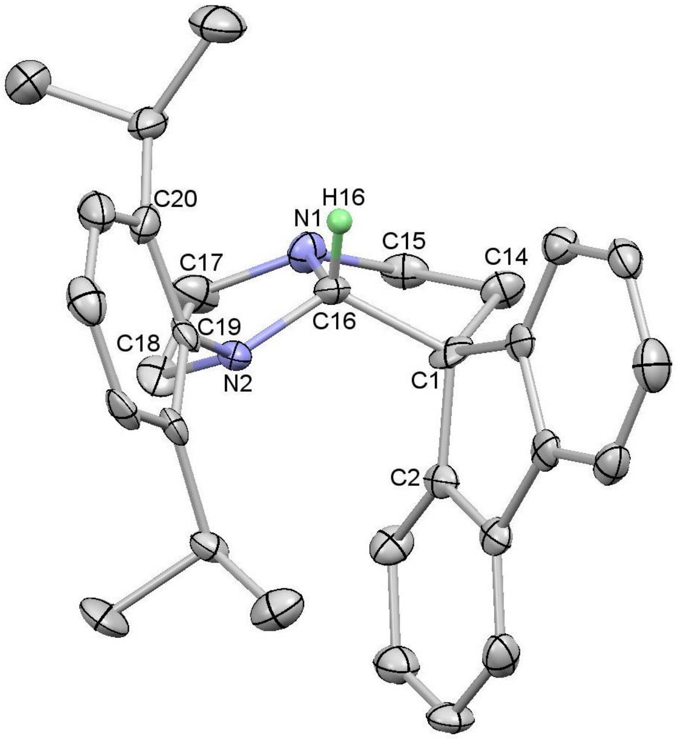

Figure S33. Molecular structure of **2** (ellipsoids at 50% probability). Only one molecule present in the asymmetric unit is shown, and all H atoms except for H16 are omitted for clarity.

Table S1 Crystallographic data for compounds **1** – **4**.

Compound	**1**	**2**	**3**	**4**
Empirical formula	$C_{30}H_{35}BF_4N_2$	$C_{30}H_{34}N_2$	$C_{36}H_{51}Li_2N_3Si_2$	$C_{39}H_{51}Li_2N_3$
Formula weight	510.41	422.59	595.85	575.70
Temperature/K	100.0	100.0	100.0	100
Crystal system	orthorhombic	monoclinic	monoclinic	monoclinic
Space group	*Pbca*	*Cc*	$P2_1/c$	$P2_1/c$
a/Å	15.2178(15)	35.165(8)	17.8401(6)	13.0403(6)
b/Å	11.8778(10)	9.280(2)	11.9822(4)	12.0336(5)
c/Å	30.409(4)	14.963(3)	17.7207(6)	22.0611(10)
α/°	90	90	90	90
β/°	90	90.931(14)	105.920(2)	92.317(3)
γ/°	90	90	90	90
Volume/Å^3	5496.6(10)	4882(2)	3642.8(2)	3459.0(3)
Z	8	8	4	4
ρ_{calc}g/cm^3	1.234	1.150	1.086	1.105
μ/mm^{-1}	0.090	0.066	0.124	0.063
F(000)	2160.0	1824.0	1288.0	1248.0
Crystal size/mm^3	0.2 × 0.12 × 0.02	0.6 × 0.35 × 0.03	0.50 × 0.35 × 0.30	0.22 × 0.22 × 0.04
Radiation	MoKα (λ = 0.71075)	MoKα (λ = 0.71073)	MoKα (λ = 0.71073)	MoKα (λ = 0.71073)
2Θ range for data collection/°	4.552 to 49.426	4.54 to 55.408	4.718 to 55.06	4.744 to 55.13
Index ranges	-17 ≤ h ≤ 17, -13 ≤ k ≤ 13, -35 ≤ l ≤ 35	-45 ≤ h ≤ 45, 0 ≤ k ≤ 12, 0 ≤ l ≤ 18	-23 ≤ h ≤ 23, -15 ≤ k ≤ 15, -22 ≤ l ≤ 23	-16 ≤ h ≤ 16, -15 ≤ k ≤ 15, -28 ≤ l ≤ 28
Reflections collected	32599	5423	45224	58481
Independent reflections	4672 [R_{int} = 0.3287, R_{sigma} = 0.1574]	5423 [R_{int} = ?, R_{sigma} = 0.1087]	8221 [R_{int} = 0.0438, R_{sigma} = 0.0410]	7934 [R_{int} = 0.0668, R_{sigma} = 0.0600]
Data/restraints/parameters	4672/0/338	5423/2/586	8221/0/415	7934/0/405
Goodness-of-fit on F^2	1.099	1.062	1.021	1.008
Final R indexes [I>=2σ (I)]	R_1 = 0.1306, wR_2 = 0.2439	R_1 = 0.0699, wR_2 = 0.1601	R_1 = 0.0423, wR_2 = 0.0959	R_1 = 0.0475, wR_2 = 0.0972
Final R indexes [all data]	R_1 = 0.2136, wR_2 = 0.2831	R_1 = 0.1132, wR_2 = 0.1890	R_1 = 0.0686, wR_2 = 0.1081	R_1 = 0.0918, wR_2 = 0.1117
Largest diff. peak/hole / e Å^{-3}	0.28/-0.35	0.42/-0.42	0.33/-0.29	0.23/-0.23

Table S1 continued Crystallographic data for compounds **5** – **7**.

Compound	**5**	**6**	**7**
Empirical formula	$C_{12}H_{23}Li_2NSi_2$	$C_{48}H_{63}N_3Na_2Si_2$	$C_{36}H_{51}K_2N_3Si_2$
Formula weight	251.37	784.17	660.17
Temperature/K	100	100.0	100.0
Crystal system	orthorhombic	monoclinic	monoclinic
Space group	*Pnma*	$P2_1/c$	$P2_1/n$
a/Å	17.7758(10)	16.8915(10)	13.0617(8)
b/Å	7.1723(3)	24.0748(13)	20.7596(14)
c/Å	12.3300(7)	11.9226(7)	13.7996(9)
α/°	90	90	90
β/°	90	106.457(2)	102.117(3)
γ/°	90	90	90
Volume/Å^3	1571.99(14)	4649.8(5)	3658.5(4)
Z	4	4	4
ρ_{calc}g/cm^3	1.062	1.120	1.199
μ/mm^{-1}	0.203	0.129	0.353
F(000)	544.0	1688.0	1416.0
Crystal size/mm^3	0.3 × 0.2 × 0.04	0.2 × 0.18 × 0.05	0.25 × 0.2 × 0.05
Radiation	MoKα (λ = 0.71073)	MoKα (λ = 0.71073)	MoKα (λ = 0.71073)
2Θ range for data collection/°	6.572 to 61.158	4.216 to 50.176	3.6 to 50.054
Index ranges	-25 ≤ h ≤ 25, -10 ≤ k ≤ 10, -17 ≤ l ≤ 17	-20 ≤ h ≤ 18, -21 ≤ k ≤ 28, -14 ≤ l ≤ 12	-15 ≤ h ≤ 15, -24 ≤ k ≤ 22, -15 ≤ l ≤ 16
Reflections collected	35485	43256	39240
Independent reflections	2577 [R_{int} = 0.0736, R_{sigma} = 0.0329]	8203 [R_{int} = 0.0873, R_{sigma} = 0.0912]	6461 [R_{int} = 0.0927, R_{sigma} = 0.0884]
Data/restraints/parameters	2577/0/98	8203/0/494	6461/0/398
Goodness-of-fit on F^2	1.035	1.012	1.017
Final R indexes [I>=2σ (I)]	R_1 = 0.0324, wR_2 = 0.0771	R_1 = 0.0540, wR_2 = 0.1058	R_1 = 0.0609, wR_2 = 0.1328
Final R indexes [all data]	R_1 = 0.0537, wR_2 = 0.0866	R_1 = 0.1161, wR_2 = 0.1263	R_1 = 0.1264, wR_2 = 0.1583
Largest diff. peak/hole / e Å^{-3}	0.35/-0.27	0.38/-0.39	1.60/-0.67

1. Bruker AXS APEX2, version 2009-5, 2009, Madison, Wisconsin, USA, Bruker AXS Inc.

2. G. Sheldrick*, Acta Crystallographica, Section A: Foundations of Crystallography* **2008**, *64*, 112-122.

3. O. V. Dolomanov, L. J. Bourhis, R. J. Gildea, J. A. K. Howard, H. Puschmann, *Journal of Applied Crystallography* **2009**, *42*, 339-341.
